# Multi-Platform Metabolomics for Fraud Detection in Spices and Botanical Food Supplements: From Fingerprint Acquisition to Decision-Grade Evidence

**DOI:** 10.3390/molecules31162938

**Published:** 2026-08-21

**Authors:** Dejan Gođevac, Stefan Ivanović, Mirjana Cvetković, Jovana Stanković Jeremić, Katarina Simić, Manuela Mandrone, Ivana Sofrenić

**Affiliations:** 1 University of Belgrade, Institute of Chemistry, Technology and Metallurgy, National Institute of the Republic of Serbia, Njegoševa 12, 11000 Belgrade, Serbia; stefan.ivanovic@ihtm.bg.ac.rs (S.I.); mirjana.cvetkovic@ihtm.bg.ac.rs (M.C.); jovana.stankovic@ihtm.bg.ac.rs (J.S.J.); katarina.simic@ihtm.bg.ac.rs (K.S.); 2 Department of Pharmacy and Biotechnology, University of Bologna, Via Irnerio, 42, 40126 Bologna, Italy; manuela.mandrone2@unibo.it; 3 University of Belgrade, Faculty of Chemistry, 11000 Belgrade, Serbia; ivanasofrenic@chem.bg.ac.rs

**Keywords:** food fraud, botanical authentication, adulteration, untargeted metabolomics, NMR spectroscopy, GC-MS, LC-HRMS, HPTLC, chemometrics, data fusion

## Abstract

Fraud in herbal medicines and botanical supplements—such as biological substitution, dilution with inert material, undeclared active pharmaceutical ingredients (APIs), and intentional mislabeling—represents a significant and underestimated threat to public health. Beyond regulatory problems, fraudulent botanical products also compromise the scientific integrity of bioactive compound research. If pharmacological or clinical studies are carried out on adulterated material without knowing it, the resulting data on safety and efficacy become unreliable, and any conclusions drawn from such data are questionable. This review critically evaluates multi-platform metabolomics as an analytical decision-making framework for botanical fraud detection. Rather than cataloging available methods, we focus on the complete analytical pipeline: study design and sample strategy, multi-platform fingerprint acquisition and processing (NMR, GC-MS, LC-HRMS, HPTLC), and chemometric modeling, validation, and decision support. Particular emphasis is placed on data fusion across platforms and the requirements for producing decision-grade evidence—analytical outputs robust enough to support market monitoring and regulatory action.

## 1. Introduction

The global market for herbal medicines and botanical dietary supplements has grown substantially over the past decade, driven by consumer demand for natural health products, increased use in integrative medicine, and expanding export markets in Asia, Europe, and North America. This growth, together with complex and often non-transparent international supply chains, has created conditions that favor economic fraud [1]. Reports from regulatory agencies such as the European Food Safety Authority (EFSA) [2], the European Commission [3], the United States Food and Drug Administration (FDA) [4], and the World Health Organization (WHO) (Refs. [5,6]) consistently show that adulteration, substitution, and mislabeling are among the most common forms of fraud in botanical food products and supplements [7,8,9,10]. Throughout this review, the term “fraud” is used in an operational analytical sense to encompass intentional adulteration, as well as other forms of chemical non-compliance that may compromise the authenticity, quality, or regulatory compliance of botanical products regardless of whether the underlying cause is deliberate or arises from negligence in the supply chain.

Fraud in this sector is not just a commercial problem. Studies on bioactive compounds, such as phytochemical profiling, pharmacological screening, or clinical investigations, can only be as reliable as the botanical material used [11].

For example, if a product labeled as *Hypericum perforatum* actually contains a substituted *Hypericum* species with a different chemical profile [12], or if various herbal products of the *Echinacea* species show significant differences in their ingredients [13], or if a *Tribulus terrestris* supplement is spiked with undeclared synthetic androgens, the scientific results become compromised in ways that may not be obvious without independent analytical verification [14,15]. For this reason, there is a strong scientific argument for rigorous and routine botanical authentication before a product is released to consumers [16].

Untargeted metabolomics, which relies on the systematic measurement of the broadest possible fraction of metabolites in a biological matrix without prior assumptions, is particularly well suited for assessing the compliance of botanical products. Compared with targeted and semi-targeted approaches, which focus on predefined analytes or chemical classes, untargeted workflows provide broader chemical coverage and may reveal unexpected deviations associated with novel adulterants, processing-related changes, or complex matrix dilution. The richness of the resulting datasets also enables the discovery of candidate diagnostic markers, whose identity and specificity should subsequently be confirmed using orthogonal analytical methods [17,18,19,20].

Multiple analytical platforms have been applied to botanical metabolomics, each offering distinct and, to some degree, complementary chemical coverage. Proton NMR spectroscopy provides a quantitative, minimally preparative, and highly reproducible global fingerprint of the solvent-soluble metabolome [21]. Gas chromatography–mass spectrometry (GC-MS) targets the volatile and semi-volatile fraction using well-established EI fragmentation libraries and offering particular sensitivity for synthetic adulterants [22,23]. Liquid chromatography–high-resolution mass spectrometry (LC-HRMS) delivers the widest chemical coverage for non-volatile, low-abundance, and structurally diverse secondary metabolites [24,25]. High-performance thin-layer chromatography (HPTLC) provides a rapid, visual, and cost-effective parallel screening option compatible with high sample throughput [23].

A critical but often underemphasized dimension of multi-platform metabolomics is the analytical decision-making process itself. How should a study be designed to distinguish fraud from genuine biological variability? How should preprocessing choices such as baseline correction, normalization, and feature selection be made and documented to ensure reproducibility? Which chemometric or machine learning strategy produces models that are genuinely predictive on new market samples, not merely well-fitted to a training set? These questions are at the heart of this review. Instead of asking “which method detects fraud?”, a question that has already been addressed in detail elsewhere [20,26], we focus on a different one: “how should an analytical chemist design, validate, and apply a multi-platform workflow that produces evidence reliable enough to support a conclusion of fraud?”

To make this discussion concrete, we draw throughout on five case studies from the authors’ own research activities: NMR metabolomics applied to oregano fraud [27], GC-MS metabolomics for oregano adulteration [28], plant metabolomics for *Allium ursinum* adulterant detection [29], HPTLC-based metabolomics for chokeberry adulteration [30], and LC-MS metabolomics applied to *Tribulus terrestris* supplement fraud in the illegal market [14].

After several analyses in our laboratories (ICTM and UBFC) of herbal dietary supplements obtained from markets, we found the prohibited drug sibutramine in “Green Coffee” declared as a 100% natural weight loss supplement [31] and the antidepressant fluoxetine in a type of “natural” weight-loss pills [32,33]. After these scandals were discovered, all counterfeit products were withdrawn from the market. Our laboratory, also, found that sildenafil and tadalafil were contained in chocolate bars (For Her And For Him) and honey (Potenza) [34], as well in capsules that were declared to be 100% natural herbal medicine (Satibo) [35]. Naltrexone, a registered medical drug that primarily serves as an antagonist in the treatment of alcohol and opiate addiction, but in low doses is also used to regulate the immune system, is found in an extract of the plant *Geissospermum vellosii* (Pao Pereira, Amazon tree), which primarily serves as a support for the immune system and complementary therapy in the fight against malignant diseases.

The specific objectives of this review are to: (i) critically evaluate multi-platform metabolomics as a decision-making framework; (ii) provide practical guidance on study design, fingerprint acquisition, and chemometric validation across NMR, GC-MS, LC-HRMS, and HPTLC; and (iii) define the “decision-grade evidence” required to translate laboratory findings into regulatory or market action.

The review is organized around a three-stage analytical workflow: (i) study design and sample strategy (Section 3 provides the fraud typology context required for rational design choices); (ii) multi-platform fingerprint acquisition and processing (Section 4); and (iii) chemometric modeling, validation, and decision support (Section 5). Section 6 discusses how laboratory evidence can be translated into real-world impact. The focus is on a tiered analytical strategy, where rapid screening methods are used to progressively select samples for more demanding confirmatory and forensic-grade analyses [36]. This section also covers the minimum evidence that laboratories should provide to support regulatory actions, publicly available databases useful for analysts, and important unresolved gaps from the analytical point of view. Topics such as blockchain-based traceability, pharmacovigilance systems, and integrated policy frameworks, although relevant to the broader problem, fall outside the analytical scope of this review and are not discussed.

## 2. Literature Selection Strategy

The literature discussed in this review was identified through iterative searches of Web of Science, Scopus, and PubMed using combinations of keywords, including “botanical fraud”, “herbal adulteration”, “untargeted metabolomics”, “multi-platform metabolomics”, “NMR”, “LC-HRMS”, “GC-MS”, “HPTLC”, and “chemometrics”, with a primary focus on publications from 2021 to early 2026. Priority was given to studies that implemented untargeted or multi-platform metabolomics workflows for fraud detection and reported sufficient methodological detail on study design, preprocessing, and modeling. The case studies drawn from the authors’ own research are used as worked examples of these general principles rather than as an exhaustive account of all available methods.

## 3. Fraud Typology and Analytical Implications

Fraud in botanical products does not take a single form. Different types of fraud produce different analytical signatures and require different detection strategies (Table 1). This section presents a concise operational typology organized not by public health consequences but by the analytical challenge that each type of fraud creates. Understanding this typology is a necessary starting point for rational study design [37].

### 3.1. Biological Substitution

Species replacement is the most common type of botanical non-compliance, whether intentional or caused by negligence in the supply chain [38]. It ranges from complete replacement of one species with another (for example, substituting one oregano species for another) to partial adulteration, where a related species is added to dilute a genuine product. From the analytical point of view, this is essentially a classification problem: can the fingerprint of an unknown sample be correctly assigned to the right species, and can partial mixtures be detected? The difficulty depends on how chemically similar the substitute is to the authentic material. Close relatives may require high-resolution platforms and multivariate modeling to distinguish, while substitution with an unrelated plant can usually be detected by visual HPTLC or simple NMR comparison.

The most prevalent kind of botanical fraud is “false arnica”: *Heterotheca inuloides* was used as a substitute for *Arnica montana* [39]. The prominent examples include substitution of commercial *Ginkgo biloba* products with *Styphnolobium japonicum* [40,41], medicinal ginger (*Zingiber officinale*) with japanese ginger (*Zingiber mioga*) [42], *Hypericum perforatum* with *Hypericum patulum* [11,12,43], and saffron (*Crocus sativus*) stigmas with the stigmas from *Carthamus tinctorius* and *Calendula officinalis* flowers. Further examples of adulteration include replacement of *Nigella sativa* black sesame seeds with seeds of *Sesamum indicum* black sesame [39], *Allium ursinum* with *Convallaria majalis* and/or *Colchicum autumnale* [44], bark of *Cinnamomum verum* cinnamon with *Cinnamomum cassia* [39,43,45], authentic *Cassia acutifolia* with *Cassia angustifolia* and *Cassia obovate*, *Saraca asoca* adulterated with *Polyalthia longifolia* [42], and *Panax ginseng* with *Panax quinquefolius* [43]. It is very common to find olive leaves (*Olea europaea*) in samples of *Juniperus* and *Tilia* in some ground herbs such as oregano and sage [39,46].

Substitution can also involve replacing a valuable material with a cheaper but distinct plant part, as in the well documented use of ground papaya (Carica papaya) seed powder to adulterate black pepper (Piper nigrum) powder [47]. In some cases, the substitute introduces its own toxicological hazard: dried Abrus precatorius root—a plant containing the highly toxic protein abrin—has been used as a substitute or covert adulterant of true licorice (Glycyrrhiza glabra) root owing to a superficially similar sweet taste and the shared common name “Indian licorice” [48].

As a complementary tool to metabolomics-based platforms, DNA barcoding—using standardized markers such as ITS2, rbcL, matK, and trnH-psbA—can independently confirm species identity in cases of biological substitution and mislabeling and has been used to detect market-wide mismatches between declared and actual species in botanical supplements [49,50]. Its diagnostic value, however, is largely confined to raw or minimally processed material: DNA degrades or is absent in extracts, tinctures, and essential oils, and it cannot detect non-biological adulterants such as undeclared synthetic APIs, since these contain no DNA [51]. For these reasons, it is best treated as an orthogonal screening layer alongside NMR, LC-HRMS, GC-MS, and HPTLC rather than a standalone solution [52].

### 3.2. Dilution with Inert Material

It should be noted that formulation excipients, including maltodextrins, cyclodextrins, and microcrystalline cellulose, are widely used in botanical supplements, and their use does not indicate fraud when properly declared on the product label. Their analytical relevance arises when they are undeclared, present in amounts inconsistent with the declared formulation, or intentionally used to dilute the botanical material. Beyond generic excipients, low-cost fillers such as corn starch and ground plant straw or husk are documented adulterants of high-value spice and herb powders, including palmarosa (*Cymbopogon martini*), oregano (*Origanum vulgare*), cumin, chili, paprika, saffron, black pepper, ginger, garlic, and mustard, where they are added to increase bulk weight at lower cost [46].

This type of non-compliance with the label is best investigated using quantitative analytical approaches. qNMR enables the absolute quantification of characteristic metabolites and can reveal deviations from expected concentration ranges. On the other hand, the detectability of inert diluents depends on their physicochemical properties and on the analytical workflow. Soluble excipients (i.e., maltodextrins) may be directly observed in the NMR spectrum, provided they are extracted under the selected analytical conditions, whereas insoluble bulking agents may primarily manifest as a decrease in metabolite concentration rather than through their own spectral signals. In this context, an important challenge is also the natural variability in marker levels across genuine samples from different growing seasons and geographic origins, which makes it necessary to have well-characterized reference ranges [38,46,53,54].

Therefore, authentication models should ideally be built using sufficiently representative reference datasets that capture this biological variability. In this context, one-class authentication approaches (see Section 5.2), in which unknown samples are evaluated according to their deviation from the chemical space defined by authentic reference materials, provide a particularly robust strategy for botanical authentication.

### 3.3. Undeclared Active Pharmaceutical Ingredients

The intentional addition of synthetic active pharmaceutical ingredients represents the highest immediate health risk, particularly in supplement categories with plausible pharmacological claims such as weight loss, sexual performance, or sports performance. Undeclared adulterants reported in botanical supplements can be grouped into several pharmacological categories.

Weight-loss and appetite-suppressant agents, including sibutramine, fenproporex, fluoxetine, lorcaserin, fenfluramine, and phenolphthalein, are the most frequently reported class, often together with metformin and furosemide [23,26,44,55,56]. Stimulants, primarily undetermined amounts of caffeine and synephrine, are also commonly found in slimming and herbal energy-boosting products [23,55]. Anabolic steroids and prohormones, including clenbuterol, selective androgen receptor modulators, tibolone, zeranol, and zilpaterol, are typically detected in supplements marketed for bodybuilding and athletic performance enhancement [46]. Analgesics and anti-inflammatory agents, such as aspirin, mefenamic acid, phenacetin, and paracetamol, have also been identified in dietary supplements, alongside antihistamines, bronchodilators/respiratory agents (theophylline, bromhexine, aminopyrine), sedatives (diazepam, chlordiazepoxide), and antidiabetic agents (glibenclamide, hydrochlorothiazide, phenytoin). Finally, in honey-based and herbal supplements marketed for sexual performance enhancement, PDE5 inhibitors and related compounds, including flibanserin, sildenafil, tadalafil, vardenafil, and their derivatives, are frequently detected [44,46,57,58].

The primary detection tool is LC-HRMS suspect screening against databases of known pharmaceutical adulterants, such as the FDA Tainted Supplements database or MassBank. The *Tribulus terrestris* case [18] is a good example: using full-scan LC-HRMS data, the authors set methods to identify multiple undeclared androgen-related compounds in the samples [55].

### 3.4. Mislabeling

Mislabeling is a common form of intentional adulteration; it involves the direct alteration of some or all of the herbal species listed in herbal supplements. Many products, capsules, tablets, soft gels, powder, or extracts often do not contain the plants listed on the declaration; for example, the following have been reported: *Curcuma longa* products that did not contain *C. longa*, *Equisetum arvense* products that did not contain *Equisetum* sp. material, supplements based on *Agathosma betulina* that did not include *A. betulina* or *A. crenulate* on the label, and products labeled as *Actaea racemosa* that did not contain *A. racemosa* material [15]. *Pausinystalia johimbe* products often contain extract or parts of *H. perforatum,* although they are labeled as yohimbe bark, its extract, or pure yohimbine [15,59]. Replacing Arabica coffee (*Coffea arabica*) with Robusta coffee (*Coffea canephora*) is one of the world’s biggest frauds [46]. The presence of withanolides has also been confirmed in the leaves of ashwagandha *Withania somnifera*; thus, root-based products have been replaced by aerial part extracts without this being declared [39]. Valerian supplements have been found to contain other botanicals (lemon balm, hops), not just valerian root or valerian root extract as stated, and valeric acid has not been found in leaf material in cases where the label indicated that a particular amount of it was contained in the product [59].

Mislabeling can occur without any adulteration—the material itself may be authentic but incorrectly labeled [50]. This is a positive identification problem rather than anomaly detection. Reliable identification requires comprehensive spectral libraries, including NMR metabolomics databases such as HMDB, BMRB, and COLMAR and MS fragmentation databases such as MzCloud, GNPS, and METLIN. NMR-based metabolomics showed that capsules claiming to contain the antimalarial artemisinin from *Artemisia* afra were mislabeled, as artemisinin is a component of *Artemisia annua* only, as well as *E. sinica*, *E. intermedia*, and *E. distachya* capsules which were sold as authentic but did not contain ephedrine, which is the main component of these plant species [53].

### 3.5. Method-Evasion Adulteration

This is analytically the most challenging type of fraud, and it involves adulterants that are specifically selected or processed to avoid detection by known analytical tests.

Very often it is necessary to detect a pure chemical marker enriched with a botanical extract [39]. Commercial turmeric root and rhizome extracts and powders are enriched with synthetic curcuminoids [39,60,61], ginkgo extracts contain spiked rutin [15,39,59], and pomegranate (*Punica granatum*) peel extracts are enriched with ellagic acid [39]. Grape seed extract, peanut skin extract, and pine bark extract are used to increase proanthocyanidin amounts in *Vaccinium macrocarpon* products [39].

As targeted quality-control methods become more widely known, sophisticated fraudsters increasingly exploit their limitations. Untargeted multi-platform metabolomics is the most robust response to this problem, since an approach that measures everything in the sample cannot be evaded by replacing one targeted analyte with another. One-class classification models, trained exclusively on authentic samples, are particularly useful in this context because they can detect any anomaly regardless of its chemical identity.

**Table 1:** Operational non-compliance typology and recommended analytical response.

Type of Non-Compliance	Analytical Challenge	Recommended Platform(s)	Key Reference(s)
Biological substitution (species replacement)	Global metabolite fingerprint differs from the authentic reference; challenge increases when closely related species share similar metabolomic profiles.	NMR, LC-HRMS; HPTLC (multi-platform preferred); DNA barcoding	[11,12,27,28,29,30,38,39,40,41,42,43,44,45,46]
Dilution with inert material (bulking agents)	Reduced relative abundance of authentic metabolites; detection depends on the physicochemical properties of the diluent and the analytical workflow.	Quantitative NMR (qNMR); LC-HRMS for targeted markers	[17,19,38,49,50,51,52,53]
Undeclared active pharmaceutical ingredients (APIs)	Untargeted detection of unexpected xenobiotics; may be at trace level; toxicological risk greatest.	LC-HRMS (suspect screening, unknown elucidation); GC-MS (volatile/synthetic APIs)	[14,16,18,23,44,55,56,57,58]
Mislabeling (authentic material, wrong label)	The material is authentic, so its profile looks normal rather than anomalous; the challenge is identifying which species it actually is via reference-library matching.	NMR + LC-HRMS; library matching; HPTLC visual identity; DNA barcoding	[15,20,27,39,44,46,53,58,59]
Method-evasion adulteration (targeted to avoid known tests)	Adulterant selected/designed to pass targeted assay; most effectively addressed through untargeted metabolomics and anomaly-detection models.	Untargeted multi-platform metabolomics; one-class anomaly models	[14,15,18,39,59,61]

## 4. Analytical Platforms: Capabilities, Complementarity, and Practical Constraints

Choosing an analytical platform for botanical authentication involves balancing chemical coverage, sensitivity, throughput, cost, infrastructure requirements, and the specific fraud type under investigation. This section reviews the four primary platforms used in the authors’ research program (NMR, GC-MS, LC-HRMS, HPTLC), addresses the untargeted-to-targeted transition required for marker elucidation, and proposes a practical decision framework for platform selection.

### 4.1. NMR-Based Metabolomics

Proton (^1^H) NMR spectroscopy has a special role in the botanical metabolomics toolkit. Its main advantages for authentication purposes are its high reproducibility and quantitative accuracy. When sample preparation, acquisition parameters, calibration, and data processing are harmonized, NMR spectra can be reliably compared across laboratories and over time. Furthermore, unlike MS-based platforms, NMR measurements are not affected by ionization efficiency or matrix-dependent ion suppression, contributing to improved analytical reproducibility. Sample preparation is minimal—usually a single extraction step followed by dilution—and the entire procedure from sample receipt to spectrum acquisition can be completed within a few hours. ^1^H NMR simultaneously detects a broad range of proton-containing metabolites that are soluble in the selected extraction medium and present above the effective detection threshold [21].

The practical limitations of NMR include lower sensitivity compared to MS-based methods (detection limits are typically in the low micromolar range), high instrument purchase costs, and throughput limitations at the highest field strengths. For these reasons, NMR is best suited for screening and batch comparison roles within a tiered analytical workflow, particularly in cases where fraud involves differences in the concentration of known metabolite classes rather than trace amounts of unusual adulterants (Box 1).

Box 1 **NMR metabolomics for oregano authentication (adapted from the authors’ own study [27]).**

One of the most commonly used spices in the Mediterranean diet is oregano. Because of its widespread use, it is often adulterated with the leaves of cheaper plants for economic gain. Oregano is frequently mixed with other plant species, such as cistus, myrtle, olive, sumac, and summer savory, or with plants whose essential oils have an aroma similar to that of oregano, such as *S. montana*, *O. majorana*, *P. anisum*, and *C. avellana*.

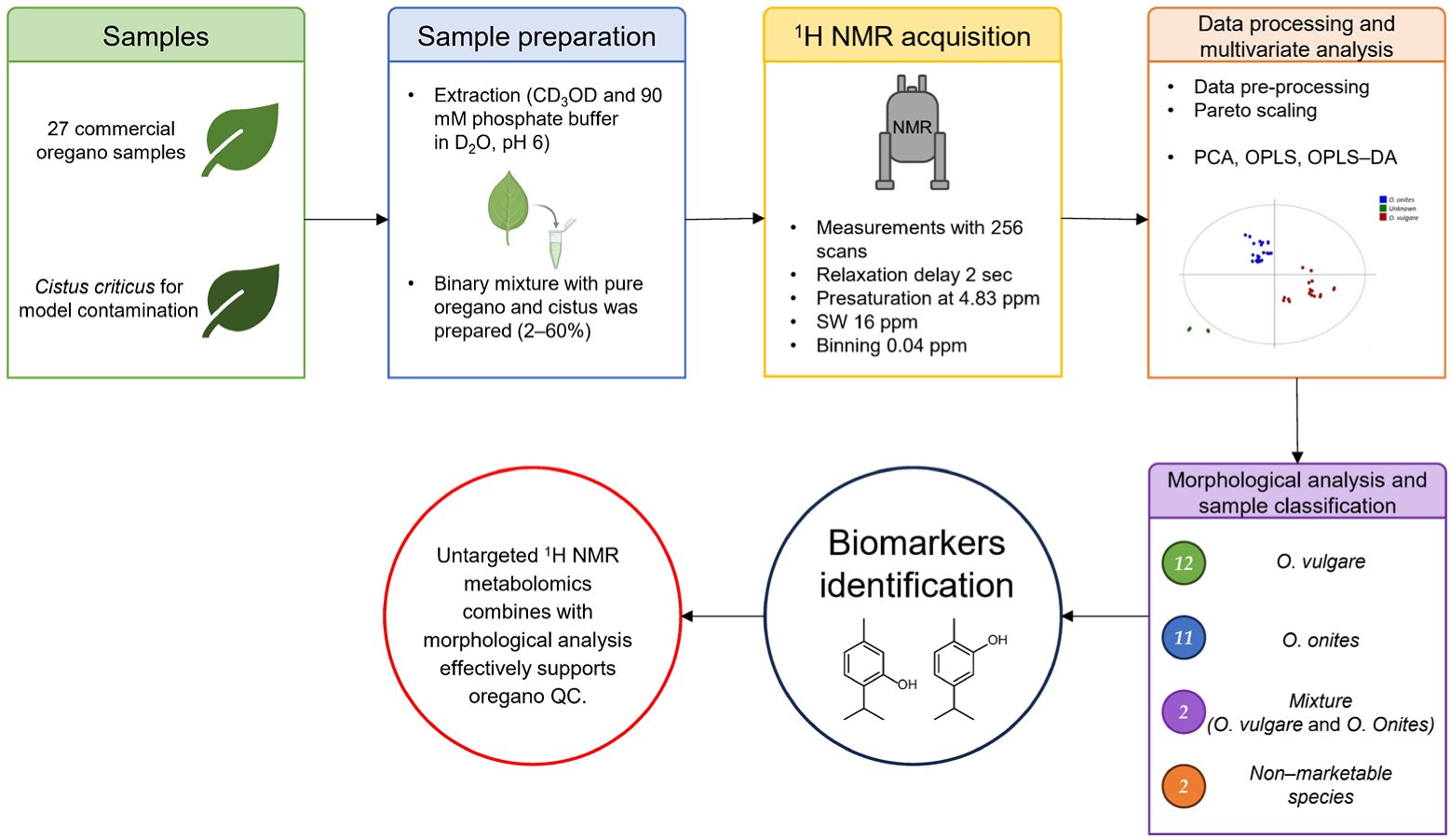

In this study, untargeted ^1^H NMR metabolomics combined with morphological analysis was applied to assess the quality of 27 oregano samples originating from Turkey, Sicily, Albania, and Peru and of samples of undeclared origin. This approach was used to detect non-marketable species, quantify adulteration, and model contamination with Cistus under a realistic industrial screening scenario.

Dried and powered samples were extracted with a 1:1 mixture of CD_3_OD and 90 mM of phosphate buffer in D_2_O (0.1% TMSP, pH 6) using ultrasonification, followed by centrifugation. Extracts were analyzed in duplicate using a 600 MHz NMR spectrometer (256 scans, 2 s relaxation delay, presaturation at δ 4.83, spectral width of 16 ppm, δ 0.04 binning). For multivariate data analysis, the data were Pareto-scaled; in addition to PCA, supervised OPLS, OPLS-DA and PLS-DA models were used.

Morphological analysis identified twelve samples as *O. vulgare*, eleven as *O. onites*, two as their mixture, and two as non-marketable species. Analysis of the OPLS-DA model, following structural characterization, identified salvianolic acid B as a biomarker of *O. vulgare*, whereas apigenin and p-cymene were characteristic of *O. onites*. Rosmarinic acid and thymol were consistently detected at comparable levels in both commer-cial oregano species (*O. vulgare* and *O. onites*), making them reliable markers of authentic commercial oregano. A marked reduction in their abundance was indicative of dilution or adulteration with non-marketable plant material. Pinitol was identified as a biomarker of adulteration with *Cistus creticus*. These findings demonstrate the value of untargeted ^1^H NMR metabolomics as a method for oregano quality control, enabling sim-ultaneous characterization of the oregano samples and detection of adulterants.

### 4.2. GC-MS-Based Metabolomics

Gas chromatography coupled to mass spectrometry (GC-MS) is the method of choice for characterizing the volatile and semi-volatile fraction of botanical matrices. Its main analytical advantage is the availability of extensive and well-validated electron ionization (EI) mass spectral libraries—NIST, Wiley, FFNSC—which allow confident compound identification in many cases without the need for reference standards. This makes GC-MS particularly useful for detecting synthetic adulterants, flavoring agents, process-derived impurities, and pesticide residues that produce characteristic EI fragmentation patterns [62,63,64,65], (Box 2).

Box 2 **GC-MS metabolomics for oregano adulteration detection (adapted from the authors’ own study [28]).**

In this study, untargeted GC-MS metabolomics was applied to identify and quantify metabolites in a total of 104 oregano samples, including eight authentic oregano samples and 96 samples artificially adulterated—with olive leaves (*O. europaea*), Venetian sumac (*C. coggigria*), and myrtle (*M. communis*)—in four different concentrations (1, 5, 10, and 20%).

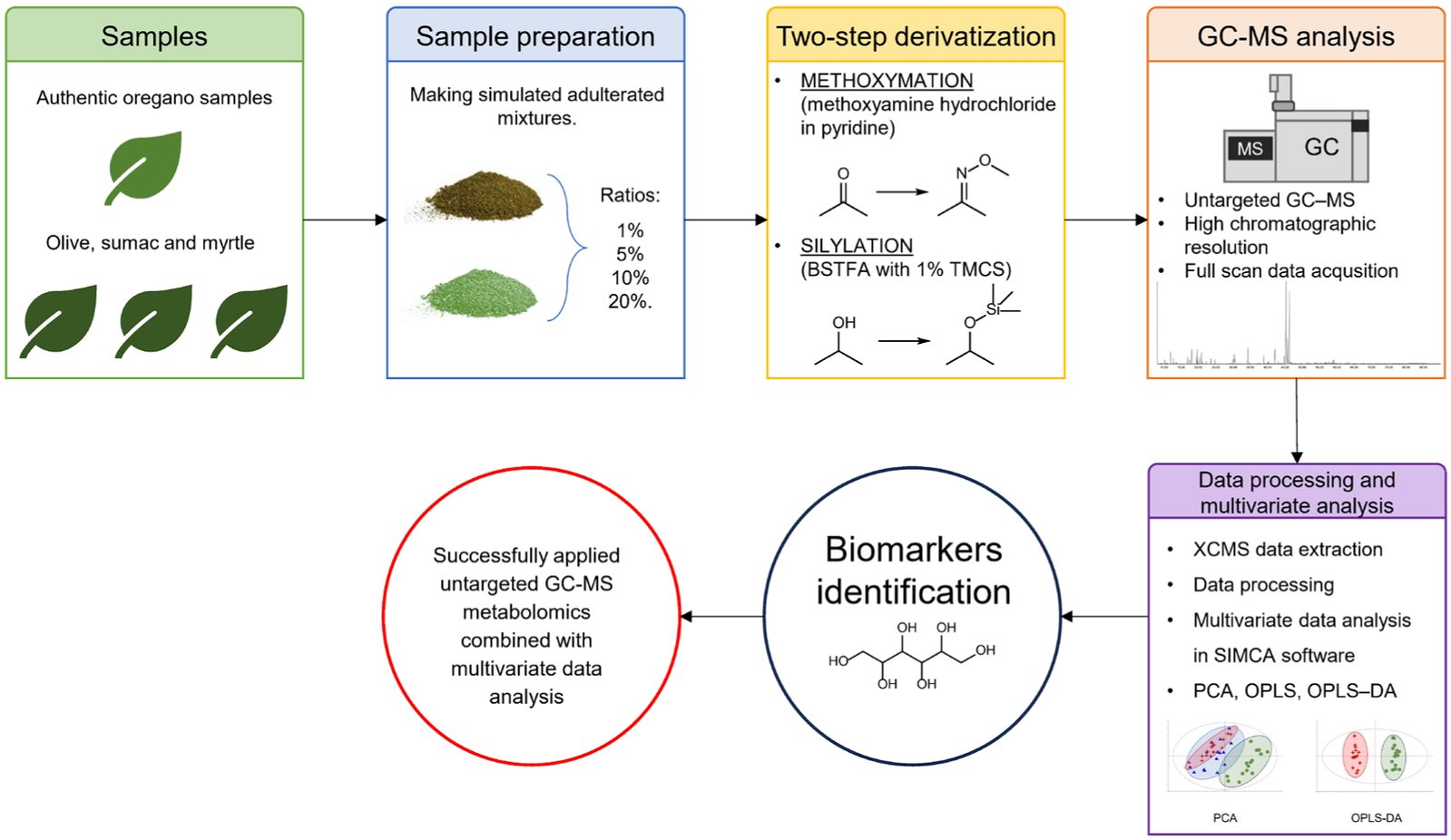

Extracts of the herbal mixtures were analyzed following a two-step derivatization procedure consisting of methoximation, followed by silylation. Metabolomic profiles were processed using multivariate analysis (PCA, OPLS, OPLS-DA) to distinguish between two types of oregano (*O. onites* and *O. vuglare*), identify markers of adulteration, and develop regression models based on the ratio of oregano and adulterants in the mixtures.

Based on the obtained results, catechol-lactate, characteristic of *O. onites*, was identified as a key discriminating metabolite for distinguishing between the two types of authentic oregano samples. Different levels of sorbitol lead to the differentiation of samples of authentic oregano and olive leaves, while shikimic and quinic acid are biomarkers of adulteration of oregano with samples of Venetian sumac. The content of fructose and quinic acid served as indicators of adulteration with myrtle leaves.

The results demonstrate that the combination of untargeted GC-MS metabolomics is a reliable approach for detecting oregano adulteration. This study is the first to apply a two-step derivatization protocol on oregano samples. Compared with the NMR approach, described in Case Study 1, GC-MS enabled the detection of a larger number of compounds because of its lower detection limits and chromatographic separation. However, GC-MS is mostly limited to volatile, derivatizable, and relatively low-molecular-weight metabolites, particularly primary metabolites such as amino acids, organic acids, sugars, etc. In contrast, NMR provides a more comprehensive representation of the extract’s chemical composition and allows the detection of a wider range of metabolites. Therefore, combining these two techniques offers a complementary strategy that maximizes metabolite coverage and provides a more comprehensive characterization of oregano samples.

The practical limitations of GC-MS include the need for derivatization of polar, non-volatile compounds, which increases sample preparation time and introduces the risk of derivatization artifacts. The method is also fundamentally limited to volatile and thermally stable analytes, and it can be difficult to distinguish authentic plant volatiles from exogenous contaminants in complex matrices [63,66,67]. Derivatization-free headspace or SPME approaches can extend the range of detectable volatiles with minimal sample preparation but at the cost of a narrower analyte window. In metabolomics workflows, GC-MS data reduction, peak alignment, and feature annotation present additional challenges, mainly because of the high degree of co-elution and in-source fragmentation that is common in complex plant extracts [68,69,70].

### 4.3. LC-HRMS-Based Metabolomics

Liquid chromatography coupled with high-resolution mass spectrometry (LC-HRMS), most commonly implemented in Orbitrap or Q-TOF configurations, represents the analytical platform offering the broadest chemical coverage among techniques used in botanical metabolomics [71]. Non-volatile, thermally labile, and structurally complex secondary metabolites, such as alkaloids, glycosides, terpenoids, phenylpropanoids, and saponins, can be analyzed without derivatization [71]. Accurate mass measurement (mass error below 5 ppm) combined with MS/MS fragmentation and computational tools, such as SIRIUS+CSI:FingerID [72], MetFrag [73], and GNPS molecular networking [74], enables high-confidence putative structural assignment without reference standards, typically corresponding to MSI Level 2–3 annotation (Section 4.5) rather than definitive Level 1 identification [17,75]. Isomeric compounds with identical formulae and similar fragmentation patterns often remain indistinguishable without orthogonal confirmation (reference standard co-elution, 2D NMR, or isolation) [75]. Full-scan acquisition strategies such as data-independent acquisition (DIA/AIF) capture all ionizable components simultaneously, allowing the dataset to be re-analyzed later as new suspect lists become available [76], (Box 3).

Box 3 **LC-MS metabolomics for *Tribulus terrestris* supplement fraud (adapted from the authors’ own study [14]).**

*Tribulus terrestris* is one of the most widely marketed botanical dietary supplements, mainly promoted for its claimed effects on muscle strength and sexual function. Its widespread use, together with the pharmacological plausibility of its claimed health benefits, makes it a frequent target for adulteration with undeclared active pharmaceutical ingredients, most commonly PDE5 inhibitors and anabolic steroids.

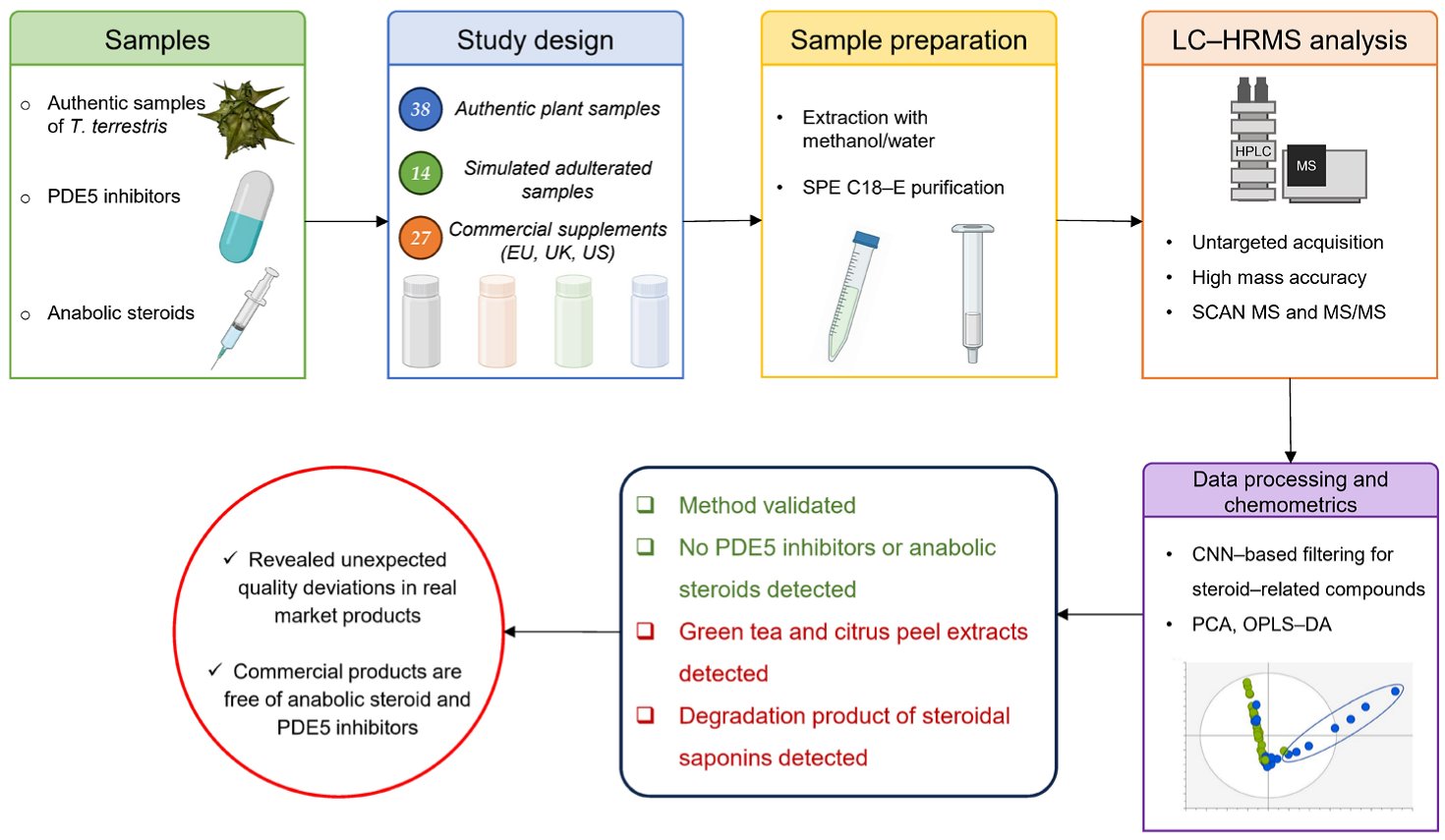

This study applied an untargeted LC-HRMS metabolomics strategy based on Orbitrap high-resolution mass spectrometry for comprehensive detection of adulterants in *T. terrestris-based* supplements. A total of 79 samples were analyzed: 38 authentic plant samples (collected in Serbia, Greece, Bulgaria, and Turkmenistan), 14 simulated adulterated samples prepared by spiking authentic material with known pharmaceutical adulterants (sildenafil, tadalafil, vardenafil, testosterone, and 4-androstene-3,17-dione), and 27 commercially available supplements obtained from European, UK, and US markets. The commercial samples were analyzed without prior knowledge of their adulteration status.

Multivariate modeling was carried out using PCA and OPLS-DA. In addition, a convolutional neural network (CNN)-based filtering tool was applied to improve the detection of steroid-related compounds. This was a methodologically important decision, since steroidal saponins are the principal bioactive components of *T. terrestris* and need to be distinguished from synthetic anabolic steroids that have similar structural features.

In the simulated adulterated samples, PDE5 inhibitors and anabolic steroids were successfully detected and confirmed. In real commercial products, no anabolic steroids or PDE5 inhibitors were found. However, the untargeted approach revealed two types of quality deviations that would not have been detected by targeted screening: (i) undisclosed green tea and citrus-derived compounds—specifically synephrine and N-methyltyramine—were identified in several products, which is consistent with the use of *Citrus aurantium* extract as an undeclared additive; (ii) spirost-4-ene-3,12-dione, a degradation product of steroidal saponins, was detected and proposed as a marker of product deterioration caused by inadequate storage or processing conditions.

This case study demonstrates two complementary strengths of untargeted LC-HRMS metabolomics: the ability to confirm suspected adulterants in controlled experiments and—equally importantly—the ability to detect unexpected quality deviations in real market samples, where the nature of potential adulteration or quality defects cannot be known in advance.

The main practical limitations of LC-HRMS are data complexity and the annotation challenge. A single analytical run produces thousands of features, and after preprocessing, which includes peak picking, deisotoping, alignment, gap-filling, and adduct annotation [77], the resulting data matrix is large and noisy. Ion suppression effects in complex matrices can cause both false positives due to interfering ions and false negatives due to suppressed authentic signals [78]. Method-specific differences related to chromatographic conditions, ionization source design, and instrument-specific mass calibration make direct comparison between laboratories difficult without careful cross-validation [76]. The annotation bottleneck, meaning that the majority of detected features remain structurally unassigned, is a recognized challenge across the entire field [79].

### 4.4. HPTLC and Hybrid Workflows

High-performance thin-layer chromatography (HPTLC) offers a rapid, visual, cost-effective, and scalable option for first-tier screening. Its main advantage is the ability to analyze many samples simultaneously on a single plate under identical conditions, a throughput advantage over chromatographic techniques that require expensive detectors. HPTLC fingerprints obtained under standardized extraction and development conditions can be directly compared to pharmacopoeial reference chromatograms, and image analysis software such as visionCATS allows automated band matching and quantification (Box 4). The low cost per sample and modest instrumentation requirements make HPTLC accessible to laboratories that cannot afford NMR or LC-HRMS infrastructure [80,81].

Box 4 **HPTLC-based metabolomics for chokeberry adulteration (adapted from the authors’ own study [31]).**

Chokeberry (*Aronia melanocarpa*) is among the richest berry species in polyphenols, anthocyanins, and other phenolic compounds, making it a target of adulteration with visually similar, sometimes toxic, species such as *Sambucus ebulus*, *Solanum nigrum*, and *Phytolacca americana*. Following documented cases of adulteration in Serbia, there is a need for reliable analytical screening methods capable of distinguishing chokeberry from these adulterants. The objectives of this study included the examination of the HPTLC profile of chokeberry fruit samples using a metabolomic approach in order to determine the botanical origin of the examined chokeberry adulterants and the identification of the most important compounds that are botanical markers responsible for classification in statistical models.

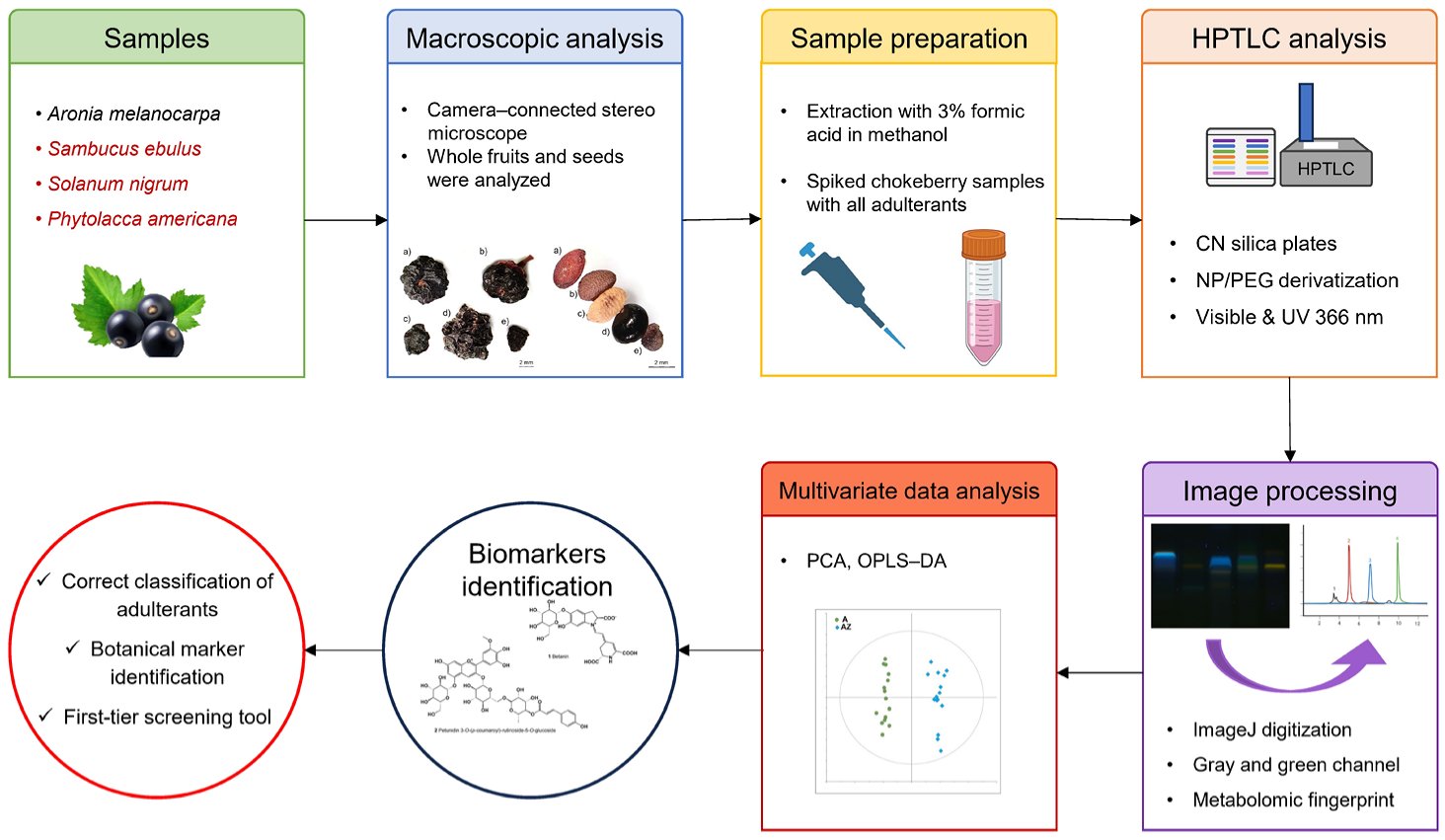

Rather than applying HPTLC as a classical single-marker identity test, this study treated the entire densitometric lane profile as a multivariate fingerprint. After developing 75 samples on five silica gel CN plates with an ethyl acetate–butanone–water–formic acid mobile phase and imaging under both visible light and UV 366 nm following NP/PEG derivatization, each chromatogram was digitized and converted into a continuous pixel intensity vector across the hR_f_ axis. This vector, representing all resolved phenolic bands simultaneously, served as the input for chemometric modeling, effectively transforming an imaging technique into an untargeted metabolomics platform.

PCA of the NP/PEG green-channel profiles explained 54.05% of variance in three components, partially resolving the five sample classes, while supervised OPLS-DA models built for each adulterant pair achieved R^2^ of 0.968–0.985 and Q^2^ of 0.955–0.972, with CV-ANOVA p-values as low as 4.77 × 10^−29^ and permutation testing ruling out overfitting. VIP analysis identified class-specific key variables: betanin (R_f_ 0.37) for pokeweed, petunidin 3-O-(p-coumaroyl)-rutinoside-5-O-glucoside (R_f_ 0.53) for black nightshade, a cluster of cyanidin sambubiosides for elderberry, and a pair of fused green bands at R_f_ 0.60–0.64 uniquely diagnostic for dwarf elderberry under NP/PEG fluorescence.

This workflow demonstrates that HPTLC-based metabolomics, coupled with image analysis, achieved high classification performance in the investigated dataset (R^2^ = 0.968–0.985, Q^2^ = 0.955–0.972), while offering practical advantages: high-throughput analysis of more than 70 samples per run, low cost, minimal solvent consumption, and direct on-plate derivatization enabling dual-detection chemical profiling. Compared to LC-MS, compound identification relies on comparison with reference standards and does not provide accurate mass for structure elucidation. However, the pattern of chromogenic bands provides complementary (orthogonal) chemical information not directly obtainable from LC-MS chromatograms or NMR spectra. Therefore, this study positions HPTLC as an effective, low-cost first-tier screening tool, with samples classified as suspect subsequently subjected to confirmatory analysis by LC-MS or NMR.

In combined workflows, HPTLC serves as a Tier 1 screening tool: samples that pass HPTLC identity confirmation continue through routine processing, while those that fail are forwarded for confirmatory Tier 2 analysis by NMR or LC-HRMS. This approach significantly reduces the workload on expensive platforms while maintaining the sensitivity and specificity of the full multi-platform workflow for samples that show anomalies. Hyphenated HPTLC–MS approaches, where individual bands are eluted directly into a mass spectrometer, provide a connection between visual screening and molecular-level identification.

### 4.5. Orthogonal Confirmation and Marker Elucidation

Untargeted metabolomics generates candidate fraud markers—features that are statistically different between authentic and adulterated samples—but statistical discrimination alone is not the same as chemical identification [82,83]. Moving from an untargeted signal to a structurally characterized and interpretable marker requires confirmation by orthogonal methods. For features detected by MS, the main tools are MS/MS fragmentation (in-source or MS2 acquisition), accurate mass-based molecular formula assignment, and computational annotation platforms [84,85]. For signals detected by NMR, 2D NMR experiments such as COSY, HMBC, HMQC, and NOESY provide connectivity and stereochemical information that goes beyond what ^1^H 1D spectra can resolve [86,87].

In practice, marker elucidation follows a sequential process: (1) statistical selection of discriminatory features, for example, by VIP score in PLS-DA or by SHAP values in random forest models; (2) putative annotation by database matching using HMDB, METLIN, or PubChem; (3) confident identification by MS/MS matching or 2D NMR assignment; and (4) optionally, isolation of the compound from the plant matrix for full structure confirmation and preparation of a reference standard. The last step is rarely performed in routine authentication work, but it is important for establishing targeted monitoring assays. The transition from untargeted to targeted analysis is therefore not simply a methodological step forward—it is also a process that generates new knowledge and contributes to a better understanding of botanical chemistry [88,89,90,91].

### 4.6. Platform Selection: A Practical Decision Framework

The question “which platform should I use?” has no context-independent answer. Platform selection is driven by a combination of factors: the suspected fraud type (from Table 1), the chemical class of markers expected, throughput requirements (number of samples per day/week), available laboratory infrastructure, per-sample cost constraints, and the eventual evidence standard required (screening vs. confirmatory vs. forensic-grade) (Table 2).

**Figure 1:**
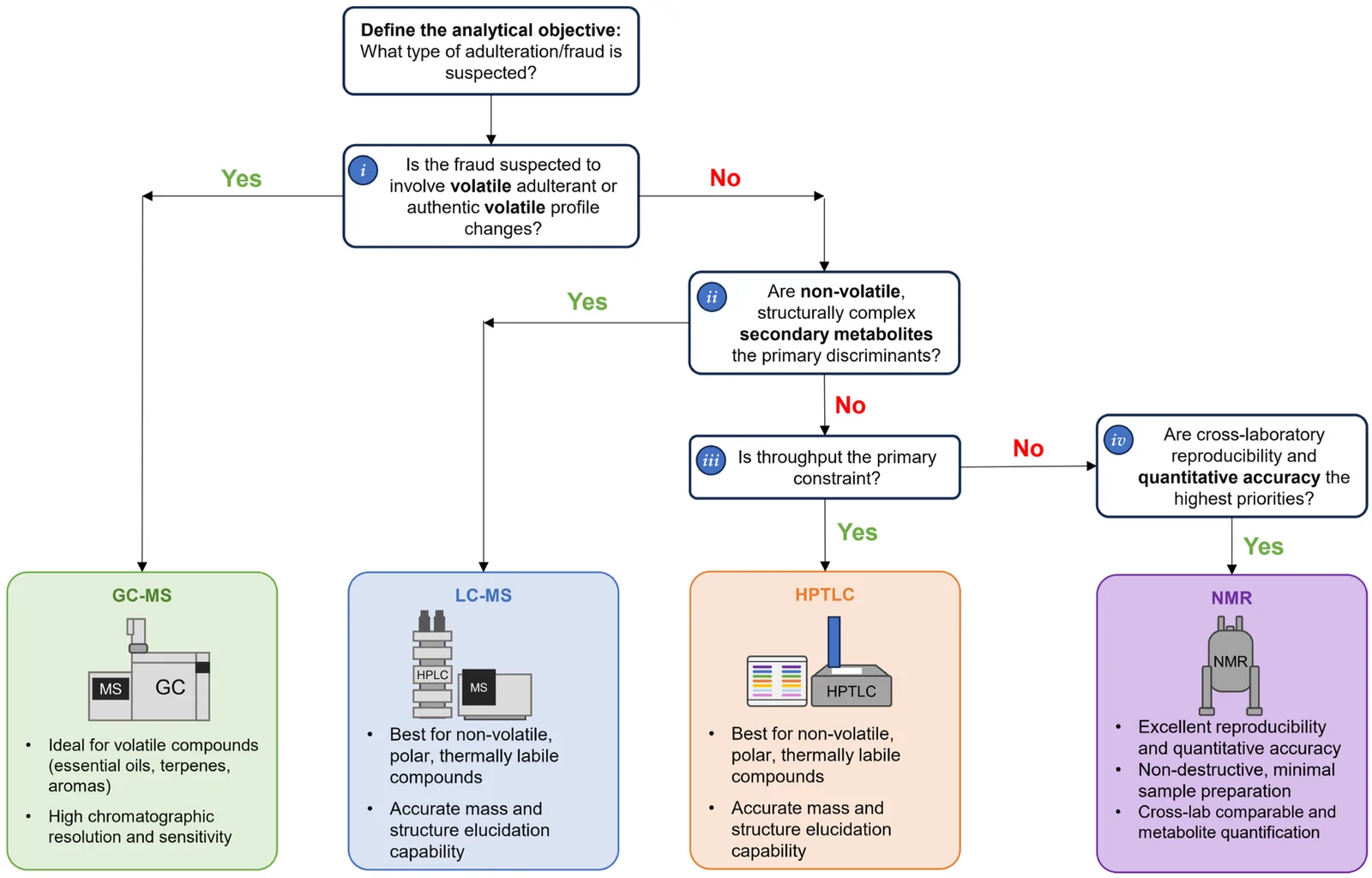
Decision tree for selecting the most appropriate analytical platform for adulteration in herbal products.

Figure 1 presents this logic as a decision tree. The key branch points are: (i) is the fraud suspected to involve volatile adulterants or alterations in the authentic volatile profile? → GC-MS; (ii) are non-volatile, structurally complex secondary metabolites the primary discriminants? → LC-HRMS; (iii) is throughput the primary constraint? → HPTLC for Tier 1 screening; (iv) are cross-laboratory reproducibility and quantitative accuracy the highest priorities? → NMR. In laboratory practice, most decision-grade workflows, when assessing authenticity, usually require at least two complementary analytical methods because, in this way, they provide a broader picture of the detected compounds and, additionally, confirm the identified markers; as a result, the probability of false negative and false positive classification is reduced.

## 5. Chemometrics, Machine Learning, and Data Fusion

Multi-platform metabolomics generates high-dimensional datasets, typically comprising hundreds to thousands of features per sample acquired across multiple analytical platforms. To translate these complex data matrices into a fraud classification outcome, whether binary or categorical, together with an associated confidence estimate, chemometric and/or machine learning approaches are required. This section provides an overview of methods ranging from classical multivariate statistical techniques to modern deep learning models, with particular emphasis on validation strategies and defining the conditions under which each approach is the most appropriate for fraud detection applications.

### 5.1. Classical Chemometrics: PCA, PLS-DA, SIMCA, OPLS-DA, OPLS

Classical chemometric methods form the basis for processing and interpretation of multi-platform metabolomic data in studies focused on fraud detection and authentication of herbal medicines and supplements. Principal component analysis (PCA) is typically the first step in such investigations, used as an unsupervised method for dimensionality reduction and visualization of major sources of variability. In herbal product authentication, PCA provides an initial overview of sample clustering, outlier detection, and assessment of consistency across samples and analytical platforms without prior class information [92,93].

For classification of predefined groups, supervised methods, such as partial least squares discriminant analysis (PLS-DA) and orthogonal partial least squares discriminant analysis (OPLS-DA), are commonly applied. These approaches use class information to identify variables responsible for group separation, with OPLS-DA additionally separating predictive and orthogonal variation, improving interpretability and marker identification (Box 5). Such models are widely used in botanical authenticity and adulteration studies, particularly for integrating data from multiple platforms [94].

Box 5 **Plant metabolomics for *Allium ursinum* adulterant detection (adapted from the authors’ own study [29]).**

*Allium ursinum* (wild garlic) is widely consumed as a food plant and dietary supplement, but its leaves share strong morphological similarities with several poisonous species that grow in the same habitats and flower at the same time. Documented poisoning incidents from accidental misidentification with *Convallaria majalis* (lily of the valley), *Vertebratum album* L. and *Colchicum autumnale* have been reported in the scientific literature. This study used *A. ursinum* as a model system to evaluate the ability of ^1^H NMR-based metabolomics to detect two poisonous adulterants: *Convallaria majalis*, which contains toxic cardiac glycosides, and *Arum maculatum*, which can cause severe allergic reactions.

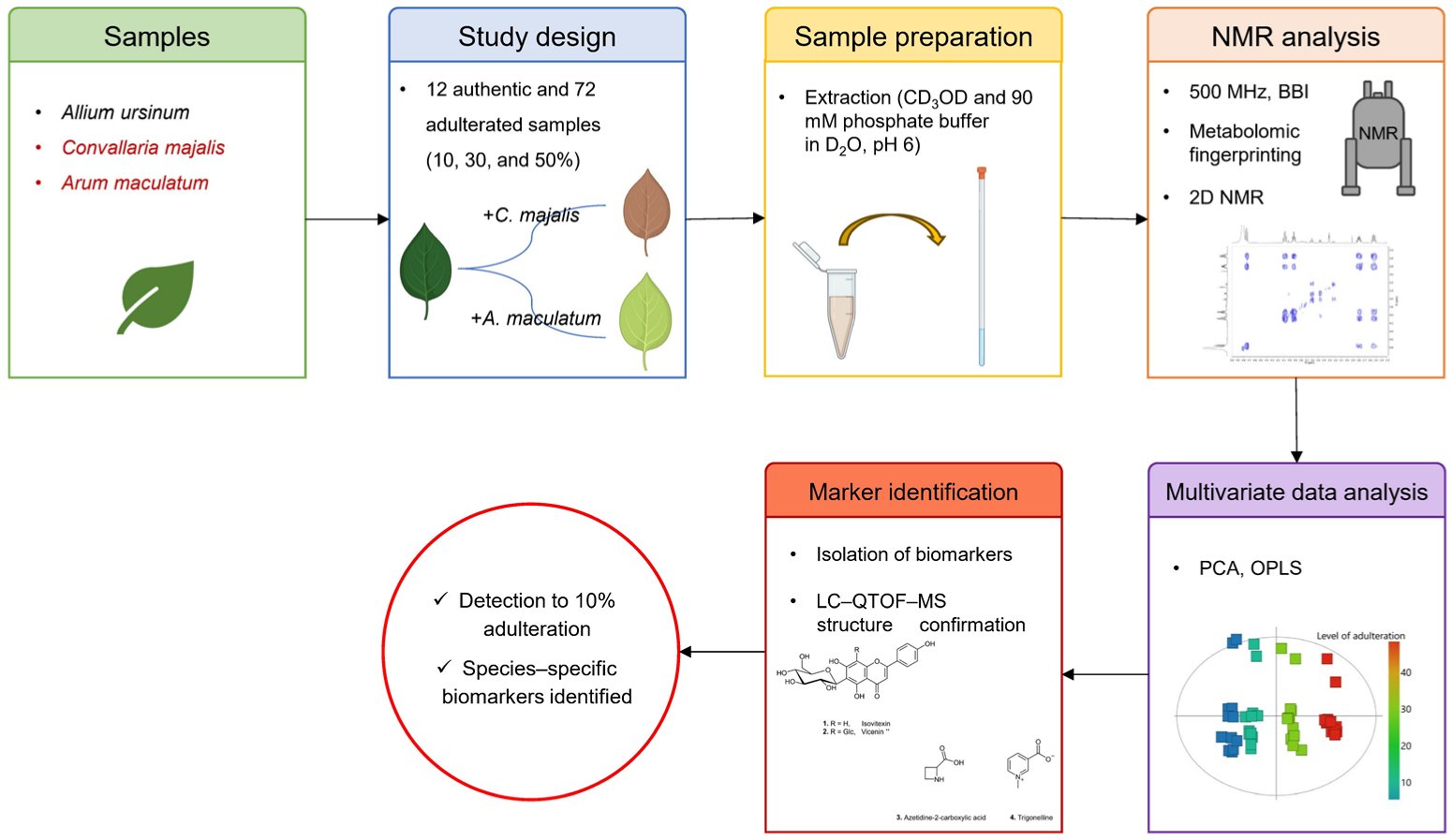

A total of 84 samples were analyzed: 12 authentic *A. ursinum* samples (2 per collection × 6 geographic locations in Serbia, ranging from lowland sites to altitudes above 1000 m) and 72 simulated adulterated samples prepared by mixing authentic *A. ursinum* leaves with *C. majalis* or *A. maculatum* at three adulteration levels (10%, 30%, and 50% *w*/*w*). Metabolomics fingerprinting was performed by ^1^H NMR spectroscopy (Bruker 500 MHz). The resulting dataset was processed by PCA followed by OPLS modeling, with VIP scores used to select the most discriminatory spectral regions. Biomarkers identified through multivariate analysis were then isolated or partially purified and their structures confirmed by 2D NMR (COSY, NOESY, HSQC, HMBC) and LC-Q-TOF-MS.

The natural variability of *A. ursinum* was itself a significant analytical challenge. PCA score plots colored by geographic origin revealed two distinct groups of authentic samples, attributed to altitude differences, where samples from above 1000 m separated clearly from those collected at lower elevations. This geographic variability in the authentic material underlines a critical problem in botanical authentication: models must be trained on a sample set that adequately represents the natural chemical space of the authentic species; otherwise, genuine high-altitude material risks being misclassified as adulterated. The six-location sampling design adopted in this study directly addressed this problem.

Despite the overlap in chemical complexity between authentic and adulterated samples visible in basic PCA plots—particularly for low-level *A. maculatum* adulteration, which required the third principal component for separation—OPLS models achieved excellent performance (R^2^ = 0.992, Q^2^ = 0.988 for C. majalis; R^2^ = 0.989, Q^2^ = 0.981 for *A. maculatum*; both with *p*-values far below 0.05 by CV-ANOVA), and adulteration was detectable down to the 10% level for both species.

Marker elucidation demonstrated the untargeted → targeted transition in practice. For *C. majalis* adulteration, four markers were identified: flavonoid C-glycosides isovitexin and vicenin II, the cyclic imino acid azetidine-2-carboxylic acid (a toxic proline antagonist), and the pyridine alkaloid trigonelline. For *A. maculatum* adulteration, isovitexin alone was identified as the diagnostic marker. Importantly, the primary toxic compounds of *C. majalis*—cardiac glycosides present at 0.1–0.5% in dry leaves—were not detected by ^1^H NMR, as their low individual concentrations and structural diversity (over 40 different glycosides identified in this species) produced signals that were obscured by the much more intense matrix signals. This illustrates an important principle: non-toxic surrogate markers can reliably indicate the presence of toxic species even when the toxic compounds themselves fall below the NMR detection threshold.

In contrast, SIMCA models each class independently and is suitable for authenticity assessment by evaluating whether a sample belongs to the reference population of authentic products rather than only enabling class separation. These methods are supported by commonly used software platforms such as SIMCA, XLSTAT, and R packages, familiar to the analytical chemistry community [93].

Key limitations of classical chemometrics include overfitting in high-dimensional, low-sample-size settings, sensitivity to preprocessing, and reduced robustness when applied to external datasets with different data distributions. Therefore, rigorous validation procedures, including k-fold or leave-one-out cross-validation and preferably independent test set validation, are essential, although not consistently applied in the botanical authenticity literature [95].

To reduce the number of variables in high-dimensional multi-platform data, three strategies are commonly used, each best suited to a particular type of model. VIP scores, calculated from PLS-DA/OPLS-DA models, are the most common choice in botanical authentication studies, since they are part of the same model used for classification and directly show which variables drive the separation between groups, as seen in the chokeberry and *Allium ursinum* case studies [29,31]. Boruta, based on random forest importance, compares each real variable against a randomized “shadow” copy of itself, which makes it a statistically robust option for noisy, non-linear data, though it requires more computation time. RFE removes the least useful variables step by step and works well with SVM-based classifiers, but in small, high-dimensional datasets it can lead to overfitting unless combined with nested cross-validation [88,89,90,91]. Based on this, we recommend VIP scores when PLS-DA/OPLS-DA models are used, Boruta for tree-based models, and RFE for SVM-based approaches with sufficiently large sample sizes.

Beyond its discriminant form, standard OPLS regression—using a continuous response variable rather than discrete class membership—is particularly useful when the goal is to predict a quantitative degree of adulteration rather than simply classify samples as authentic or adulterated. For example, an OPLS model built with the percentage of adulterant (independently quantified by microscopy) as the response variable outperformed discriminant approaches for predicting adulteration level in oregano, while also improving interpretability of the underlying spectral variables and enabling prediction on new samples [27].

### 5.2. Anomaly Detection and One-Class Models

One of the challenges in botanical fraud detection stems from the fact that authentic samples can be readily collected, characterized, and used to construct representative training sets, whereas the range of possible adulterants is virtually unlimited. Consequently, classifiers trained on known examples of authentic and counterfeit products are often unable to recognize novel forms of fraud that are not represented in the training set. This limitation motivates the use of one-class models, which define the boundaries of the authentic sample space and identify any significant deviation from this space as a potential anomaly, without requiring prior knowledge of all possible types of adulteration [96].

SIMCA, discussed in the previous section, is one of the most widely used examples of one-class modeling. Other approaches include one-class support vector machines (OC-SVM), isolation forest, autoencoder-based anomaly detection, and one-class partial least squares (OC-PLS). Each of these methods has its own advantages and limitations. In terms of sample requirements, SIMCA and OC-PLS can be developed using relatively small datasets, whereas autoencoder-based approaches generally require substantially larger datasets and greater computational resources. These methods are particularly valuable in situations where new or previously unknown forms of adulteration may emerge and where it is impractical to model all potential fraud scenarios in advance [96,97].

A major challenge associated with one-class approaches is the reliable definition of the boundaries of the authentic sample space from a finite and inherently incomplete training set. Therefore, it is essential that the reference set of authentic samples is sufficiently representative and capture the variability associated with geographical origin, growing season, post-harvest processing and storage conditions.

### 5.3. Machine Learning Classifiers: RF, SVM, CNN

When the dataset is sufficiently large, typically comprising 50–100 or more samples per class, machine learning methods can outperform classical chemometric approaches in terms of both predictive accuracy and robustness. In the context of fraud detection and authentication of herbal medicinal products, three machine learning classifiers are particularly widely used: random forest (RF) [98], support vector machine (SVM) [99], and convolutional neural networks (CNNs) [100]. RF is an ensemble method based on a large number of decision trees, in which the final classification is determined by majority voting. It is robust against overfitting, relatively insensitive to noise and outliers, and well suited for high-dimensional datasets such as NMR, LC-MS, and IR spectroscopic profiles. SVM, a margin-based classifier, is particularly effective in situations involving a relatively small number of samples and a large number of variables, which are common characteristics of metabolomic datasets [98]. By employing kernel functions, such as linear, radial basis function, or polynomial kernels, SVM constructs optimal decision boundaries in high-dimensional feature spaces. In contrast, CNNs and other deep learning approaches enable the automatic extraction of hierarchical features directly from raw or minimally processed data, including NMR (FID) signals and chromatographic profiles. These models are especially suitable for spectroscopic data, which can be represented as one-dimensional signals. Although CNNs can reduce the need for explicit feature extraction, they generally require substantially larger training datasets, often exceeding 500 samples, and are computationally demanding [100]. Collectively, these methods constitute an important component of modern supervised classification frameworks in botanical authentication, with their selection depending on dataset size, data availability, and problem complexity. They are frequently combined with classical chemometric techniques, such as PCA and PLS-DA, in multi-platform integration workflows, where RF and SVM are primarily used for the classification of predefined groups, whereas CNNs are employed to identify complex patterns in spectroscopic data [101].

The main risk associated with the application of machine learning methods in botanical authentication is optimistic bias resulting from improper model validation. Cross-validation schemes that allow information leakage from the test set into the training set, for example when data scaling or feature selection is performed on the entire dataset prior to data splitting, can lead to unrealistically high estimates of model accuracy. Independent external test sets, consisting of samples that were not used at any stage of model development, represent the most reliable approach for evaluating model performance [102]. Therefore, studies lacking external validation should be interpreted with caution, regardless of the reported accuracy values.

### 5.4. Data Fusion Across Platforms

Data fusion across platforms refers to the integration of data obtained from multiple analytical techniques into a unified modeling framework. In practice, this involves combining data generated by NMR, LC-MS, IR, HPLC analysis, or various sensor-based approaches in order to obtain a more comprehensive representation of the investigated sample than that provided by each method individually. The aim is not only dimensionality reduction but also appropriate scaling, normalization, and integration of variables to preserve relevant information while minimizing noise and method-specific limitations [99].

There are different levels of data fusion. Early fusion combines raw or partially processed data prior to modeling, mid-level fusion integrates features extracted from each platform, whereas late fusion combines the outputs of separate models built for each platform. Low-level fusion is conceptually the simplest approach. However, it can easily be dominated by the platform contributing the largest number of features, most often LC-HRMS, if appropriate scaling and dimensionality reduction are not applied. For this reason, mid-level fusion (multiblock approaches) is often considered the most balanced solution, as it preserves the internal structure of each dataset while exploiting their complementarity [103]. In contrast, high-level fusion is more robust to missing data, since classification can still be performed even if one platform is unavailable, and it does not capture potential synergistic interactions between features derived from different platforms. In practice, mid-level and late fusion are often easier to implement when platforms differ in dimensionality, scale, and data structure.

In chemometrics and metabolomics, this approach is particularly important because different platforms capture different layers of chemical information. For example, NMR typically provides a comprehensive and highly quantitative overview of major metabolites, whereas LC-MS is more sensitive to trace and secondary metabolites, and IR spectroscopy enables rapid acquisition of a global fingerprint profile. When these data sources are combined, improved discrimination between authentic and counterfeit samples can be achieved, along with enhanced detection of subtle differences related to geographical origin or processing, as well as increased model robustness [103].

For instance, NMR-based datasets typically contain a relatively limited number of highly reproducible variables (approximately 300–500 features), whereas LC-HRMS datasets often include a substantially larger number of variables, ranging from 5000 to 50,000 detected features. This disparity can introduce significant bias in integrated models, as high-dimensional platforms may dominate the variance structure and thereby drive model construction, potentially suppressing informative but lower-dimensional data sources. To mitigate the impact of differences in dimensionality across analytical platforms, appropriate scaling, normalization, and weighting strategies are required to ensure balanced contributions from each platform. In this context, multiblock and integrative approaches such as the MOCA (multiblock orthogonal component analysis) framework represent an effective solution, as they enable separate modeling of each data block while preserving their complementarity. In this way, MOCA enables controlled integration of heterogeneous datasets, reduces the impact of dimensional imbalance, and improves the interpretability of classification models [104].

The combination of NMR and MS platforms has proven to be a particularly powerful strategy in modern metabolomics, integrating the high quantitative reproducibility of NMR with the broad chemical coverage of mass spectrometry. A 2025 review in *Molecules* on NMR–MS data fusion strategies provides a comprehensive methodological framework with direct relevance to botanical authentication [105]. In addition, a study on *Tribulus terrestris* [14] demonstrated that the three-dimensional structure of LC-MS data, when analyzed using deep learning methods, contains additional discriminatory information for distinguishing authentic and counterfeit samples that cannot be fully captured by conventional two-dimensional feature-matrix-based approaches.

The experience of our research group in multi-platform metabolomics and the integration of heterogeneous datasets provide a strong foundation for the development and application of advanced approaches in authentication and fraud detection [14,27,28,29,30].

For unknown adulterants specifically, high-level fusion tends to work better, since it combines the outputs of separate one-class models built for each platform. This way, if only one platform detects an anomaly, the signal is not diluted by the other platforms’ normal-looking data. Mid-level fusion is still a good choice when the goal is to characterize a known adulterant that produces related signals across several platforms, but it is less effective for anomalies that show up on only one platform.

### 5.5. Model Validation, Interpretability, and Deployment

There is a significant difference between classification models developed for research purposes and those suitable for routine market surveillance. Models developed in a research setting are typically trained and evaluated on samples collected within a defined time period and under specific analytical conditions. However, models intended for practical application must maintain their accuracy when exposed to new samples originating from different geographical regions, cultivation seasons, and supply chains. This issue, known as dataset shift (or concept drift), leads to a degradation in model performance over time [106]. In botanical authentication, this variability arises from seasonal and geographical factors, as well as differences in experimental conditions, including different laboratories and instruments. As a result, botanical samples exhibit high intrinsic variability, since their metabolomic profiles change depending on harvesting season, geographical origin, and post-harvest processing. This inherent variability may lead to overfitting to the training set and limited applicability to independent new data, where authentic samples may be incorrectly classified as adulterated due to natural differences [106].

In the development of chemometric and machine learning models for botanical authentication, three key aspects can be distinguished: model validation, interpretability, and practical deployment.

Model validation represents a critical step in ensuring robustness and applicability to independent datasets. Commonly used methods include k-fold cross-validation, leave-one-out cross-validation, and, ideally, external validation on an independent test set. These approaches provide a more realistic estimate of model performance and reduce the risk of overfitting. Given the high natural variability of botanical samples discussed in the previous section, rigorous validation is essential to ensure model generalization across different data distributions and real-world conditions. Therefore, models intended for practical use must be validated on external, independent datasets that capture natural sample variability, accompanied by transparent reporting of performance metrics and detailed documentation of preprocessing steps to ensure reproducibility [102].

Beyond classification accuracy, an important aspect is model interpretability, i.e., the ability to identify chemical markers responsible for classification. From a scientific perspective, methods such as VIP scores and OPLS-DA loading plots, as well as machine learning-based methods, feature importance measures, such as random forest feature importance and SHAP analysis, that enable the linking of model decisions to specific metabolites [103]. From a practical standpoint, interpretable models provide higher confidence and regulatory value, as classification supported by identified markers carries stronger evidential weight than black-box approaches.

Model deployment in real-world conditions involves integration into quality control systems and regulatory inspection of botanical products. Key challenges include robustness to missing data, scalability, and stability of performance on new market samples. In this context, late fusion approaches are particularly suitable, as they enable robust classification even in the absence of individual analytical platforms, while standardized preprocessing and continuous data drift monitoring ensure long-term model stability [99].

According to AOAC (Association of Official Analytical Chemists) [107] guidelines and the current literature, minimum reporting standards include: a representative training set, external validation on independent samples, an appropriate cross-validation protocol, standard performance metrics (sensitivity and specificity), model interpretability (VIP, SHAP, or feature importance), and transparent documentation of preprocessing steps and continuous monitoring of model performance over time for drift detection [102].

## 6. From Laboratory Evidence to Real-World Impact

Robust analytical evidence for botanical fraud is a necessary but not sufficient condition for real-world impact, which happens when analytical proof becomes traceable, reproducible, and compatible with regulatory evidence standards [98]. This section addresses the analytical dimensions of translating laboratory results into actionable intelligence: tiered analytical strategy, minimum evidence documentation, publicly available databases as analytical tools, and critical unresolved gaps from the analyst’s perspective. The main focus represents evidence readiness: can the obtained analytical data be transferred into a regulatory and forensic context? Regulatory policy frameworks, pharmacovigilance networks, and digital traceability systems are relevant to the broader problem but are outside the analytical scope of this review. For the decision grade evidence, it is necessary to use validated methods, clear decision boundaries, transparent QC and clearly categorized identification levels [108].

### 6.1. A Tiered Analytical Strategy for Market Monitoring

**Figure 2:**
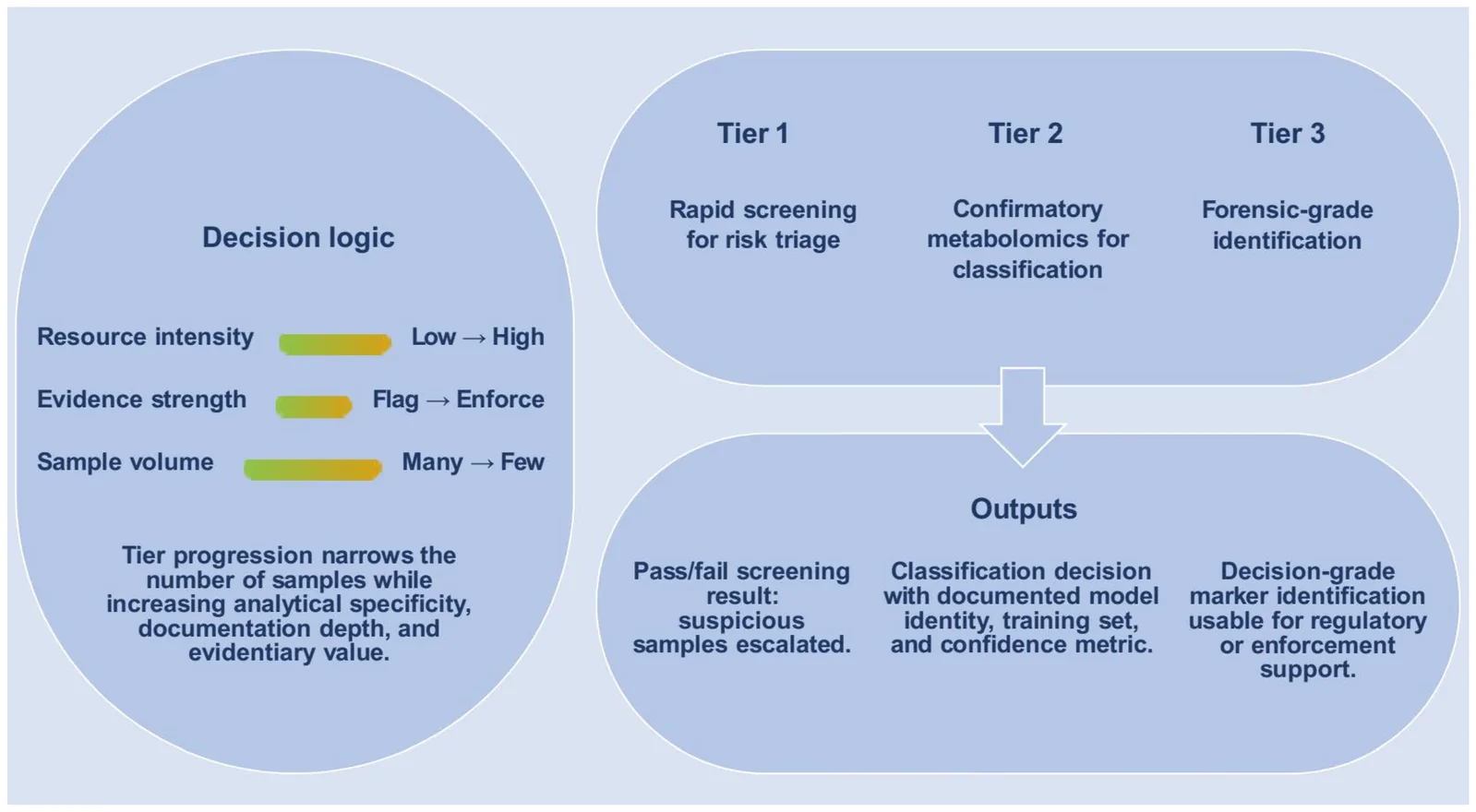
Tiered analytical strategy for market monitoring of botanical products.

A tiered analytical strategy allows regulatory and quality control laboratories with limited resources to maintain broad market coverage without requiring the most expensive analytical platforms for every sample. The main criteria focus on fitness for regulatory purposes and resource requirements, resulting in clear distinctions from ordinary target and untargeted approaches, which primarily consider metabolome coverage [109]. In the context of botanical authentication, three tiers can be distinguished (Figure 2).

Tier 1—rapid screening for risk triage: This tier includes HPTLC identity fingerprinting, NIR spectroscopy, or simple ^1^H NMR comparison against reference spectra [110]. These methods are high-throughput and low-cost, with high sensitivity, and their primary purpose is to flag suspicious samples for further investigation rather than to provide definitive conclusions about fraud. For Tier 1 methods to be reliable, robust pharmacopoeia-validated reference chromatograms or spectra are needed, and the methods should be calibrated against known adulterant types for the commodity of interest.

Tier 2—confirmatory metabolomics for classification and pattern recognition: Samples that do not pass Tier 1 screening, or those from supply chains with an elevated risk of fraud, proceed to untargeted metabolomics workflows using NMR and/or LC-HRMS [111]. Classification models trained on validated reference collections are then applied, and the output is a classification decision with associated confidence metrics [90]. Tier 2 authentication requires a standardized sampling protocol (in which geographical, seasonal, and process diversity are addressed), defined QC protocols (QC pool samples, blanks), and transparent preprocessing documentation (normalization, scaling, correction of the batch effect). The final results represent clearly defined decision rules, such as conformity index and probability of class membership, rather than only suggested differences between samples. Samples where adulteration is suspected are forwarded to Tier 3.

Tier 3—forensic-grade identification: This tier involves structural identification of fraud-associated markers at the highest achievable confidence level (Metabolomics Standards Initiative—metabolite identification guidelines, MSI Levels 1–2), supported by reference standard comparison, 2D NMR, or isolation and full structure elucidation. Evidence produced at this tier is suitable for supporting enforcement actions. The authors’ study on *Tribulus terrestris* supplements [14] illustrates Tier 3 capability: accurate-mass LC-HRMS with MS/MS confirmation was used to identify undeclared active pharmaceutical ingredients in commercial samples.

### 6.2. Minimum Analytical Evidence Package for Regulatory Support

A complete evidence package supporting an analytical conclusion of non-compliance or suspected fraud should include, at minimum: (i) a unique batch identifier and sample traceability documentation for the suspect sample; (ii) documentation of the sampling protocol, including location, date, sample size, and storage conditions; (iii) the Tier 1 screening result, with a pass or fail conclusion, a method reference, and the reference chromatogram or spectrum used; (iv) the Tier 2 metabolomics result, including the identity and version of the model, the composition of the training set, and the classification output with an associated confidence metric; (v) Tier 3 confirmation for any specific markers identified, including accurate mass, MS/MS fragmentation match, and, where available, comparison with a reference standard; and (vi) complete documentation of the preprocessing and analysis pipeline in sufficient detail for independent reproduction. The proposed standard format of the analytical evidence checklist is presented in Table 3.

This package does not, in itself, constitute a regulatory decision which involves legal, jurisdictional, and policy considerations that go beyond the scope of the laboratory. However, assembling it proactively and in a standardized format substantially increases the value of analytical findings for regulatory authorities and makes cross-laboratory comparison and inter-laboratory validation much easier.

### 6.3. Publicly Available Resources and Databases

Several publicly available databases are useful in practice for analytical chemists working on botanical authentication. The FDA Tainted Dietary Supplements Database lists products in which the FDA has found undeclared pharmaceutical ingredients, which is very useful both for building suspect lists for LC-HRMS screening and for deciding which product categories to prioritize in monitoring campaigns. (Public Notifications–Health Fraud (Health Fraud Product Database/List of Tainted Supplements, 2025 update [112]), FDA, Avoiding Products Contaminated with Hidden Ingredients, 2025 [112].) The EU RASFF (Rapid Alert System for Food and Feed) [113] portal provides notifications of alerts related to botanical products across EU member states and can serve as an early-warning resource for emerging fraud types. Pharmacopoeial monographs such as the *European Pharmacopoeia* (12th edition, Council of Europe), *USP* (*United States Pharmacopeia and National Formulary*, USP 48–NF 43), and *Chinese Pharmacopoeia* (*Pharmacopoeia of the People’s Republic of China*, 2025 edition) provide validated reference chromatograms, chemical marker specifications, and accepted analytical methods for a large number of botanical commodities.

For metabolomics-specific resources, the Human Metabolome Database (HMDB) [114], METLIN [115], MeRy-B [116], MassBank [117], Global Natural Products Social Molecular Networking (GNPS) [74], and COLMAR [118] for NMR provide spectral reference data for compound identification. The Natural Products Atlas [119] and the Dictionary of Natural Products (DNP) [120] support the annotation of plant-derived secondary metabolites. The Metabolomics Workbench [121] and METABOLIGHTS [122,123] repositories allow deposition and retrieval of metabolomics datasets, which supports inter-laboratory comparison and model validation [124]. However, no single database provides complete coverage of the chemical diversity of botanical products. Consequently, confident compound annotation and botanical authentication often require interrogation of multiple complementary databases and orthogonal analytical evidence. Moreover, botanical authentication datasets still remain underrepresented compared to clinical metabolomics.

### 6.4. Gaps and Challenges from the Analytical Perspective

Despite significant methodological progress, several critical gaps limit the transition of multi-platform metabolomics from research to routine analytical practice for botanical authentication.

Lack of standardized reference datasets: Botanical authentication models are only as good as the reference collections on which they are trained. Currently, no internationally accepted, publicly deposited reference datasets exist for most commercially important botanical commodities [125]. Different research groups use their own in-house collections, making cross-study comparison and model validation nearly impossible. This is arguably the single most important infrastructure gap in the field.

Limited inter-laboratory validation: Method validation in botanical metabolomics rarely extends beyond the originating laboratory. Instrument-specific spectral characteristics, regional differences in authentic material, and variations in preprocessing choices between laboratories mean that all contribute to models that perform well internally but degrade significantly when applied to samples processed in a different laboratory [110]. AOAC [126] and EURACHEM [127] guidelines for method validation apply in principle, but their practical implementation for untargeted metabolomics workflows remains largely uncharted territory.

The annotation bottleneck: In LC-HRMS untargeted metabolomics, the majority of detected features—often 70–90%—remain structurally unassigned at the end of a typical workflow [128,129]. Classification models built on unassigned features may be accurate but are not interpretable in chemical terms. Advances in computational annotation (SIRIUS and ZODIAC for molecular formula prediction [72,130], CSI:FingerID for structural assignment [131], CANOPUS for class prediction [132], GNPS molecular networking [74]) are reducing this bottleneck, but confident structural identification still requires considerable expert effort and complete structure elucidation for novel plant secondary metabolites.

The natural variability vs. adulteration discrimination problem. Perhaps the most intellectually fundamental challenge is distinguishing genuine biological variation (geographic, seasonal, ecotypic) from chemical changes induced by adulteration [125]. A model trained on oregano populations grown under similar soil and climate conditions showed that different populations, even when cultivated in controlled conditions, can form different chemotypes [133,134].

Addressing this requires not only broad reference collections spanning authentic variability but also careful attention to feature selection. This means that only features that are variable between authentic and adulterated samples, while stable within authentic populations, should contribute to the classification.

## 7. Conclusions and Future Perspectives

Metabolomics has the potential to transform botanical authentication from a marker-based discipline into a comprehensive assessment of the global chemical phenotype of a formulation. Rather than relying on a limited number of predefined compounds, untargeted analytical approaches enable the detection of unexpected chemical deviations associated with species substitution, dilution, undeclared pharmaceutical ingredients, processing-related modifications, and other forms of non-compliance. As datasets grow beyond 100 samples, as identified in Section 5.3, deep learning approaches such as convolutional neural networks are likely to become increasingly viable alternatives to classical chemometrics, particularly for capturing complex, non-linear multi-platform fusion patterns. In parallel, real-time and portable platforms, such as handheld NMR and ambient ionization mass spectrometry, hold promise for extending Tier 1 screening (Figure 1) into field and market-surveillance settings.

However, metabolomics alone is not sufficient to generate reliable evidence. Throughout this review, we have highlighted that robust botanical authentication depends on the entire analytical workflow, including representative sampling, careful experimental design, standardized sample preparation, transparent data preprocessing, appropriate chemometric modeling, rigorous validation, and orthogonal confirmation of key findings. Multi-platform analytical strategies further strengthen the reliability of the conclusions by providing complementary chemical information that cannot be captured by a single technique. A major standardization gap remains across platforms: harmonized reference databases and interlaboratory validation protocols for NMR, LC-HRMS, GC-MS, and HPTLC are still lacking, limiting the transferability of models developed in individual laboratories to routine multi-site quality control.

An equally important challenge is the natural biological variability of botanical materials. Differences in geographical origin, genotype, cultivation conditions, harvesting time, processing, and storage may substantially affect metabolomic fingerprints and should therefore be adequately represented in reference datasets. Future authentication models will increasingly rely on well-curated libraries of authentic reference materials and one-class authentication strategies capable of distinguishing genuine biological variability from true chemical anomalies. Expanding public data infrastructure is essential to this effort: suspect-screening libraries should be broadened and kept current with emerging adulterants, and the field would benefit from open-access, curated spectral repositories for authentic botanical reference materials, enabling smaller laboratories to build representative one-class models without independently collecting large reference sample sets.

Future progress will also depend on closer integration between analytical “decision-grade evidence,” as defined throughout this review, and the specific thresholds regulatory agencies require to act on a finding of fraud. This review has intentionally focused on the analytical side of this pipeline; translating validated multi-platform metabolomic outputs into evidentiary standards accepted by regulators remains an open and underexplored area that will require closer collaboration between analytical chemists and regulatory scientists.

Taken together, these findings indicate that robust botanical authentication is unlikely to rely on a single analytical platform. Instead, progress will be driven by the integration of complementary analytical techniques, advanced chemometric approaches, harmonized data-sharing practices, and standardized reporting frameworks capable of transforming metabolomic fingerprints into transparent, reproducible, and decision-grade analytical evidence suitable for routine quality control and regulatory application.

## Figures and Tables

**Table 2:** Comparative overview of analytical platforms for botanical metabolomics.

Platform	Strengths	Limitations	Best Suited For	Throughput	Cost Tier ^1^
^1^H NMR	Quantitative, reproducible, minimal prep, high analytical reproducibility, non-destructive	Low sensitivity, high instrument cost, signal overlap in complex mixtures	Screening, global fingerprinting, batch comparison, dilution detection, qNMR, structural elucidation	Medium–high	High (instrument)
GC-MS	EI libraries, synthetic adulterant detection, volatile coverage, high sensitivity and resolution	Derivatization needed for polars, limited to volatile/semi-volatile	Volatile or derivatizable analytes and adulterants, essential oil authentication	Medium	Low–medium
LC-HRMS	Widest chemical coverage, non-volatile and thermolabile compounds, suspect screening, structural elucidation of unknowns	Complex data processing, annotation bottleneck, ion suppression, method-specific biases, inter-platform variability, high computational and data storage requirements	Non-volatile secondary metabolites, undeclared APIs, structural elucidation	Low–medium	High
HPTLC	Parallel analysis, visual fingerprint, low cost and minimal infrastructure requirements, pharmacopoeial compatibility	Limited resolution, subjective band interpretation without software, semi-quantitative	Tier 1 screening, identity confirmation, high-throughput pre-selection	High	Low

^1^ Cost tier refers primarily to instrumentation and infrastructure requirements rather than per-sample analytical costs.

**Table 3:** Proposed standardized analytical evidence checklist.

Part	Description	Completed	Not Applicable	Notes
Section A				
A1	Unique batch identifier (internal ID, manufacturer lot)	☐	☐	
A2	Sample source documented (manufacturer, distributor, retail, online)	☐	☐	
A3	Chain-of-custody record (dates, persons, transfer steps)	☐	☐	
A4	Purchase/shipment documentation archived	☐	☐	
Section B				
B1	Sampling location recorded (country, city)	☐	☐	
B2	Sampling date recorded	☐	☐	
B3	Sample size and composite sampling strategy documented	☐	☐	
B4	Storage conditions recorded (temperature, light, humidity, container)	☐	☐	
B5	Time from collection to analysis documented	☐	☐	
B6	Deviations from sampling SOP documented	☐	☐	
Section C				
C1	Tier 1 technique specified (HPTLC, NIR, ^1^H NMR, etc.)	☐	☐	
C2	Method reference cited (pharmacopoeia, SOP, the literature)	☐	☐	
C3	Pass/fail decision recorded with criteria	☐	☐	
C4	Reference chromatogram/spectrum archived	☐	☐	
C5	Suspect sample chromatogram/spectrum archived and annotated	☐	☐	
C6	Analytical deviations from the reference profile documented (e.g., missing, additional, or significantly altered features)	☐	☐	
Section D				
D1	Metabolomics platform specified	☐	☐	
D2	Sample preparation protocol documented	☐	☐	
D3	Model identity and version recorded	☐	☐	
D4	Training set composition described (n, diversity)	☐	☐	
D5	Validation strategy reported (cross-validation, external set, metrics)	☐	☐	
D6	Classification decision for suspect sample recorded	☐	☐	
D7	Confidence metric/conformity index reported	☐	☐	
Section E				
E1	List of fraud-associated markers compiled	☐	☐	
E2	MSI confidence level assigned to each marker (Level 1/2/annotation)	☐	☐	
E3	Accurate mass data archived (m/z, error)	☐	☐	
E4	MS/MS fragmentation match documented (library/in-house, score)	☐	☐	
E5	Retention time comparison with reference standard documented	☐	☐	
E6	Orthogonal confirmation recorded (2D NMR, UV–Vis, isolation)	☐	☐	
E7	Reference standard source, purity, and traceability documented	☐	☐	
Section F				
F1	Preprocessing steps listed (alignment, baseline correction, normalization, scaling)	☐	☐	
F2	Batch effect correction and QC strategy described	☐	☐	
F3	Data analysis methods and software versions recorded	☐	☐	
F4	Model parameters and decision thresholds documented	☐	☐	
F5	Location of raw data files and metadata specified	☐	☐	
F6	Availability of scripts/workflows (repository, version) documented	☐	☐	

## Data Availability

The original contributions presented in this study are included in the article. Further inquiries can be directed to the corresponding author.

## References

[1] Han Q., Erasmus S.W., Elliott C.T., van Ruth S.M. (2022). A sense of ginger fraud: Prevalence and deconstruction of the China-European Union supply chain. npj Sci. Food.

[2] Rapid Alert System for Food and Feed (RASFF)—Consumer Window. https://webgate.ec.europa.eu/rasff-window/screen/consumers.

[3] European Commission Food Fraud Network (FFN) Monthly Report, March 2026. https://food.ec.europa.eu/document/download/39c8523f-e57b-4f39-a33e-0711d21886a2_en?filename=ff_ffn_monthly-report_202603.pdf.

[4] Medication Health Fraud Notifications. U.S. Food and Drug Administration (FDA). https://www.fda.gov/drugs/medication-health-fraud/medication-health-fraud-notifications.

[5] WHO Expert Committee on Specifications for Pharmaceutical Preparations (2017). Fifty-First Report.

[6] World Health Organization (WHO) (2017). WHO Global Surveillance and Monitoring System for Substandard and Falsified Medical Products.

[7] Frequency of Fraud in Herbs and Spices. Knowledge for Policy, European Commission. https://knowledge4policy.ec.europa.eu/food-fraud-quality/frequency-fraud-herbs-spices_en.

[8] Adulteration of Herbs and Spices. Knowledge for Policy, European Commission. https://knowledge4policy.ec.europa.eu/projects-activities/adulteration-herbs-spices_en#more_information_and_links.

[9] (2025). Commission Finds Fraud and Potential Safety Issues in Cinnamon on the EU Market. Joint Research Centre, European Commission. https://joint-research-centre.ec.europa.eu/jrc-news-and-updates/commission-finds-fraud-and-potential-safety-issues-cinnamon-eu-market-2025-09-24_en.

[10] Health Fraud Product Database. U.S. Food and Drug Administration (FDA). https://www.fda.gov/consumers/health-fraud-scams/health-fraud-product-database.

[11] Alyas A.A., Aldewachi H., Aladul M.I. (2024). Adulteration of herbal medicine and its detection methods. Pharmacogn. J..

[12] Raclariu A.C., Paltinean R., Vlase L., Labarre A., Manzanilla V., Ichim M.C., Crisan G., Brysting A.K., de Boer H. (2017). Comparative authentication of *Hypericum perforatum* herbal products using DNA metabarcoding, TLC and HPLC-MS. Sci. Rep..

[13] Raclariu A.C., Tebrencu C.E., Ichim M.C., Ciuperca O.T., Brysting A.K., de Boer H. (2018). What’s in the box? Authentication of *Echinacea* herbal products using DNA metabarcoding and HPTLC. Phytomedicine.

[14] Gođevac D., Stanković Jeremić J., Cvetković M., Simić K., Sofrenić I., Ljujić J., Popović L., Gašić U., Hoang Y.-N., Huan T. (2025). LC-MS-Based Metabolomics for Detecting Adulteration in *Tribulus terrestris*-Derived Dietary Supplements. Food Chem. X.

[15] Ichim M.C., Booker A. (2021). Chemical authentication of botanical ingredients: A review of commercial herbal products. Front. Pharmacol..

[16] Cohen P.A. (2014). Hazards of hindsight—Monitoring the safety of nutritional supplements. N. Engl. J. Med..

[17] Sumner L.W., Amberg A., Barrett D., Beale M.H., Beger R., Daykin C.A., Fan T.W., Fiehn O., Goodacre R., Griffin J.L. (2007). Proposed minimum reporting standards for chemical analysis: Chemical Analysis Working Group (CAWG) Metabolomics Standards Initiative (MSI). Metabolomics.

[18] Tugizimana F., Piater L., Dubery I. (2013). Plant metabolomics: A new frontier in phytochemical analysis. S. Afr. J. Sci..

[19] Wishart D.S. (2019). Metabolomics for investigating physiological and pathophysiological processes. Physiol. Rev..

[20] Liu Z., Yang M.Q., Zuo Y., Wang Y., Zhang J. (2022). Fraud detection of herbal medicines based on modern analytical technologies combined with chemometrics approach: A review. Crit. Rev. Anal. Chem..

[21] Lolli V., Caligiani A. (2024). How nuclear magnetic resonance contributes to food authentication: Current trends and perspectives. Curr. Opin. Food Sci..

[22] Samanta K., Nath P., Patel R., Singh G., Jain A., Nandi G. (2024). Advances of GC-MS in the determination of adulterants in dietary supplements. Proceedings of the E3S Web of Conferences.

[23] Gheorghiu O.R.C., Ciobanu A.M., Guțu C.M., Dănilă G.-M., Nițescu G.V., Rohnean Ș., Baconi D.L. (2025). Detection of adulterants in herbal weight loss supplements. J. Mind Med. Sci..

[24] Sakai T., Tanaka M., Azuma Y., Sakamoto Y., Tagami T., Asada A., Doi T. (2026). Simultaneous identification of 50 illegal adulterants in dietary supplements using high-performance liquid chromatography–single quadrupole mass spectrometry. J. Pharm. Biomed. Anal..

[25] Anwar M.S., Khan A., Khan I., Khan S.A., Ahmad L., Kaleem W.A., Mahzari A., Al-Megrin W.A.I., Almatroudi A., Allemailem K.S. (2023). Evaluation of marketed herbal medicines for the simultaneous estimation of steroidal adulterants using FTIR and RP-HPLC-UV. Microchem. J..

[26] Dipadharma R.H.F., Prayugo I.J., Layandro O.A. (2021). Recent analytical method for detection of chemical adulterants in herbal medicine. Molecules.

[27] Mandrone M., Marincich L., Chiocchio I., Petroli A., Gođevac D., Maresca I., Poli F. (2021). NMR-based metabolomics for frauds detection and quality control of oregano samples. Food Control.

[28] Ivanović S., Mandrone M., Simić K., Ristić M., Todosijević M., Mandić B., Gođevac D. (2021). GC-MS-based metabolomics for the detection of adulteration in oregano samples. J. Serb. Chem. Soc..

[29] Ivanović S., Simić K., Lekić S., Jadranin M., Vujisić L., Gođevac D. (2022). Plant metabolomics as a tool for detecting adulterants in edible plant: A case study of *Allium ursinum*. Metabolites.

[30] Ivanović S., Gođevac D., Ristivojević P., Zdunić G., Stojanović D., Šavikin K. (2023). HPTLC-based metabolomics approach for the detection of chokeberry (*Aronia melanocarpa* (Michx.) Elliott) adulteration. J. Herb. Med..

[31] Vučković I., Karajić A., Vujisić L., Jadranin M., Vajs V. Zelena kafa^®^—Istine i zablude. Proceedings of the 48th Meeting of the Serbian Chemical Society.

[32] Book of Abstracts. Symposium, Institute of Chemistry, Technology and Metallurgy (IHTM), University of Belgrade. https://ihtm.bg.ac.rs/images/novosti/simpozijum/Apstrakti.pdf.

[33] Đavo iz Kineske Laboratorije. Vreme. https://vreme.com/vreme/djavo-iz-kineske-laboratorije/.

[34] Vuković G., Pantić-Palibrk V., Đukić M., Vujisić L., Anđelković B. Assessment of synthetic adulterants in dietetic products on Serbian market. Proceedings of the 8th International Symposium on Recent Advances in Food Analysis.

[35] (2016). Spektroskopija na Studentskom Trgu. Faculty of Chemistry, University of Belgrade. https://www.chem.bg.ac.rs/muzej/2016-Spektroskopija/Spektroskopija_na_Studentskom_trgu.pdf.

[36] Mück F., Scotti F., Mauvisseau Q., Thorbek B.L.G., Wangensteen H., de Boer H.J. (2024). Three-tiered authentication of herbal traditional Chinese medicine ingredients used in women’s health provides progressive qualitative and quantitative insight. Front. Pharmacol..

[37] Haji A., Desalegn K., Hassen H. (2023). Selected food items adulteration, their impacts on public health, and detection methods: A review. Food Sci. Nutr..

[38] Ndlovu P.F., Magwaza L.S., Tesfay S.Z., Mphahlele R.R. (2022). Destructive and rapid non-invasive methods used to detect adulteration of dried powdered horticultural products: A review. Food Res. Int..

[39] Gafner S., Blumenthal M., Foster S., Cardellina J.H., Khan I.A., Upton R. (2023). Botanical ingredient forensics: Detection of attempts to deceive commonly used analytical methods for authenticating herbal dietary and food ingredients and supplements. J. Nat. Prod..

[40] Bampali E., Germer S., Bauer R., Kulić Ž. (2021). HPLC-UV/HRMS methods for the unambiguous detection of adulterations of *Ginkgo biloba* leaves with *Sophora japonica* fruits on an extract level. Pharm. Biol..

[41] Grazina L., Amaral J.S., Costa J., Mafra I. (2021). Tracing *Styphnolobium japonicum* (syn: *Sophora japonica*) as a potential adulterant of ginkgo-containing foods by real-time PCR. J. Food Compos. Anal..

[42] Kaur R., Choudhary T., Kaur R., Garg M., Chauhan N.S., Jain A., Baldi A. (2025). Adulteration in herbal medicines: A comprehensive review on types, detection, and health impacts. Pharmacogn. Res..

[43] Orhan N., Gafner S., Blumenthal M. (2025). Ginseng adulteration across global markets and evaluation of commercial product authenticity. Nat. Prod. Commun..

[44] Steuer C., Raeber J. (2025). A story of falsification and authentication: Authenticity control of phytopharmaceuticals and herbal remedies. Eur. J. Pharm. Sci..

[45] Riswanto F.D.O., Windarsih A., Lukitaningsih E., Rafi M., Fadzilah N.A., Rohman A. (2022). Metabolite fingerprinting based on 1H-NMR spectroscopy and liquid chromatography for the authentication of herbal products. Molecules.

[46] Velázquez R., Rodríguez A., Hernández A., Casquete R., Benito M.J., Martín A. (2023). Spice and herb frauds: Types, incidence, and detection: The state of the art. Foods.

[47] Orrillo I., Cruz-Tirado J.P., Cardenas A., Oruna M., Carnero A., Barbin D.F., Siche R. (2019). Hyperspectral imaging as a powerful tool for identification of papaya seeds in black pepper. Food Control.

[48] Kumar P., Lone J.F., Gairola S. (2022). Comparative macroscopic and microscopic characterization of raw herbal drugs of *Abrus precatorius* L. and *Glycyrrhiza glabra* L.. Pharmacogn. Res..

[49] Mahima, Sunil Kumar K.N., Rakhesh K.V., Rajeswaran P.S., Sharma A., Sathishkumar R. (2022). Advancements and future prospective of DNA barcodes in the herbal drug industry. Front. Pharmacol..

[50] Harris C.M., Kim D.Y., Jordan C.R., Miranda M.I., Hellberg R.S. (2024). DNA barcoding of herbal supplements on the US commercial market associated with the purported treatment of COVID-19. Phytochem. Anal..

[51] Raclariu-Manolică A.C., Mauvisseau Q., de Boer H.J. (2023). Horizon scan of DNA-based methods for quality control and monitoring of herbal preparations. Front. Pharmacol..

[52] Nazar N., Saxena A., Sebastian A., Slater A., Sundaresan V., Sgamma T. (2024). Integrating DNA barcoding within an orthogonal approach for herbal product authentication: A narrative review. Phytochem. Anal..

[53] Momtaz M., Bubli S.Y., Khan M.S. (2023). Mechanisms and health aspects of food adulteration: A comprehensive review. Foods.

[54] Jyoti T.P., Mitra S., Chandel S., Singh R. (2025). Essentials of qNMR: Navigating adulteration and quality control challenges across food, herbal medicines and beyond. Pharmacol. Res. Nat. Prod..

[55] Vinothkanna A., Dar O.I., Liu Z., Jia A.-Q. (2024). Advanced detection tools in food fraud: A systematic review for holistic and rational detection method based on research and patents. Food Chem..

[56] Esposito G., Sciuto S., Martello E., Pezzolato M., Bozzetta E. (2023). Disclosing frauds in herbal food supplements labeling: A simple LC-MS/MS approach to detect alkaloids and biogenic amines. J. Food Prot..

[57] Supposedly Herbal Products. Swissmedic, Swiss Agency for Therapeutic Products. https://www.swissmedic.ch/swissmedic/en/home/humanarzneimittel/market-surveillance/medicinal-products-from-the-internet/warnings-related-to-medicinal-products-from-the-internet/warnung_pflanzlichen_produkten.html.

[58] Phan D.T.A., Kongkaew C., Heinrich M., Dao T.C.M., Vo T.H. (2025). From ‘traditional’ remedies to ‘modern’ supplements: A systematic review and meta-analysis of pharmaceutical adulteration in weight-loss natural products. Front. Pharmacol..

[59] Pujol C., Danoun S., Biasini G., Retailleau E., Masson J., Balayssac S., Gilard V. (2024). Benchtop NMR coupled with chemometrics: A workflow for unveiling hidden drug ingredients in honey-based supplements. Molecules.

[60] Pawar R.S., Handy S.M., Cheng R., Shyong N., Grundel E. (2017). Assessment of the authenticity of herbal dietary supplements: Comparison of chemical and DNA barcoding methods. Planta Med..

[61] You H., Gershon H., Goren F., Xue F., Kantowski T., Monheit L. (2022). Analytical strategies to determine the labelling accuracy and economically-motivated adulteration of “natural” dietary supplements in the marketplace: Turmeric case study. Food Chem..

[62] McGlynn D.F., Yee L.D., Garraffo H.M., Geer L.Y., Mak T.D., Mirokhin Y.A., Tchekhovskoi D.V., Jen C.N., Goldstein A.H., Kearsley A.J. (2025). New library-based methods for nontargeted compound identification by GC-EI-MS. J. Am. Soc. Mass Spectrom..

[63] Frolova N., Orlova A., Popova V., Bilova T., Frolov A. (2025). Gas chromatography–mass spectrometry (GC-MS) in the plant metabolomics toolbox: Sample preparation and instrumental analysis. Biomolecules.

[64] Wenio I., Derewiaka D., Majewska E., Bartosiewicz I., Ryszkowska E. (2024). Development of a rapid method for simultaneous determination of pesticides in plant oil using GC-MS/MS. Appl. Sci..

[65] Zhu H., Zhu D., Sun J. (2023). Application of GC-IMS coupled with chemometric analysis for the classification and authentication of geographical indication agricultural products and food. Front. Nutr..

[66] Frolova N., Bilova T., Silinskaia S., Orlova A., Gurina A., Frolov A. (2026). Gas chromatography–mass spectrometry (GC-MS) in the plant metabolomics toolbox: GC-MS in multi-platform metabolomics and integrated multi-omics research. Int. J. Mol. Sci..

[67] Verpoorte R., Kim H.K., Choi Y.H. (2022). Trivialities in metabolomics: Artifacts in extraction and analysis. Front. Mol. Biosci..

[68] Zhou W., Pawliszyn J. (2024). Perspective on SPME-MS: Green and high-performance methods for rapid screening. Anal. Chim. Acta.

[69] Ovbude S.T., Sharmeen S., Kyei I., Olupathage H., Jones J., Bell R.J., Powers R., Hage D.S. (2024). Applications of chromatographic methods in metabolomics: A review. J. Chromatogr. B.

[70] Ghafari N., Sleno L. (2024). Challenges and recent advances in quantitative mass spectrometry-based metabolomics. Anal. Sci. Adv..

[71] Aydöğan C. (2020). Recent advances and applications in LC-HRMS for food and plant natural products: A critical review. Anal. Bioanal. Chem..

[72] Dührkop K., Fleischauer M., Ludwig M., Aksenov A.A., Melnik A.V., Meusel M., Dorrestein P.C., Rousu J., Böcker S. (2019). SIRIUS 4: A rapid tool for turning tandem mass spectra into metabolite structure information. Nat. Methods.

[73] Ruttkies C., Schymanski E.L., Wolf S., Hollender J., Neumann S. (2016). MetFrag relaunched: Incorporating strategies beyond in silico fragmentation. J. Cheminform..

[74] Wang M., Carver J.J., Phelan V.V., Sanchez L.M., Garg N., Peng Y., Nguyen D.D., Watrous J., Kapono C.A., Luzzatto-Knaan T. (2016). Sharing and community curation of mass spectrometry data with Global Natural Products Social Molecular Networking. Nat. Biotechnol..

[75] Schymanski E.L., Jeon J., Gulde R., Fenner K., Ruff M., Singer H.P., Hollender J. (2014). Identifying small molecules via high resolution mass spectrometry: Communicating confidence. Environ. Sci. Technol..

[76] Tsugawa H., Cajka T., Kind T., Ma Y., Higgins B., Ikeda K., Kanazawa M., VanderGheynst J., Fiehn O., Arita M. (2015). MS-DIAL: Data-independent MS/MS deconvolution for comprehensive metabolome analysis. Nat. Methods.

[77] Nasiri A., Jahani R., Mokhtari S., Yazdanpanah H., Daraei B., Faizi M., Kobarfard F. (2021). Overview, consequences, and strategies for overcoming matrix effects in LC-MS analysis: A critical review. Anal. Methods.

[78] Clark T.N., Houriet J., Vidar W.S., Kellogg J.J., Todd D.A., Cech N.B., Linington R.G. (2021). Interlaboratory comparison of untargeted mass spectrometry data uncovers underlying causes for variability. J. Nat. Prod..

[79] da Silva R.R., Dorrestein P.C., Quinn R.A. (2015). Illuminating the dark matter in metabolomics. Proc. Natl. Acad. Sci. USA.

[80] Mukherjee P.K. (2019). High-performance thin-layer chromatography (HPTLC) for analysis of herbal drugs. Quality Control and Evaluation of Herbal Drugs.

[81] Mulaudzi N., Combrinck S., Vermaak I., Joubert E., Viljoen A. (2022). High performance thin layer chromatography fingerprinting of rooibos (*Aspalathus linearis*) and honeybush (*Cyclopia genistoides*, *Cyclopia intermedia* and *Cyclopia subternata*) teas. J. Appl. Res. Med. Aromat. Plants.

[82] Couto C.d.C., Chávez D.W.H., Oliveira E.M.M., Freitas-Silva O., Casal S. (2024). SPME-GC-MS untargeted metabolomics approach to identify potential volatile compounds as markers for fraud detection in roasted and ground coffee. Food Chem..

[83] Tan H.R., Zhou W. (2024). Metabolomics for tea authentication and fraud detection: Recent applications and future directions. Trends Food Sci. Technol..

[84] Wawrzyniak R., Ruperez F.J., Godzień J.B. (2023). Editorial: Advances and challenges in untargeted metabolomics. Front. Mol. Biosci..

[85] Domingo-Almenara X., Montenegro-Burke J.R., Benton H.P., Siuzdak G. (2018). Annotation: A computational solution for streamlining metabolomics analysis. Anal. Chem..

[86] Domenici V. (2024). Detection of adulterations and contaminations in food products by NMR. The Environment in a Magnet.

[87] Prakash C., Mahar R. (2025). Chemical profiling and quality assessment of food products employing magnetic resonance technologies. Foods.

[88] Wu Y., Wang Y. (2025). An improved metabolomics workflow enables untargeted data acquisition and targeted data analysis using liquid chromatography–mass spectrometry. J. Proteome Res..

[89] Zhang B., Li S., Liang Z., Wei Y., Dong J., Wen H., Guo L., Hao X., Zhang Y. (2025). The application and perspective of NMR and MS based strategies for functional compounds mining in medicinal and dietary plants. Food Sci. Hum. Wellness.

[90] Borges R.M., Teixeira A.M. (2024). On the part that NMR should play in mass spectrometry metabolomics in natural products studies. Front. Nat. Prod..

[91] Gerothanassis I.P. (2026). NMR spectroscopy in complex mixture analysis and structure elucidation of natural products: Rethinking the need for separations. Separations.

[92] Feng Y., Zhu X., Wang Y. (2025). Application of spectroscopic technology with machine learning in Chinese herbs from seeds to medicinal materials: The case of genus Paris. J. Pharm. Anal..

[93] De Angelis D., Summo C., Pasqualone A., Faccia M., Squeo G. (2024). Advancements in food authentication using soft independent modelling of class analogy (SIMCA): A review. Food Qual. Saf..

[94] Ballabio D., Consonni V. (2013). Classification tools in chemistry. Part 1: Linear models. PLS-DA. Anal. Methods.

[95] Rodionova O.Y., Oliveri P., Malegori C., Pomerantsev A.L. (2024). Chemometrics as an efficient tool for food authentication: Golden pillars for building reliable models. Trends Food Sci. Technol..

[96] Harnly J. (2023). Botanical authentication using one-class modeling. J. AOAC Int..

[97] Harnly J. (2025). One-class modeling for verification of botanical identity: A review. Front. Pharmacol..

[98] Alum E.U., Manjula V.S., Uti D.E., Echegu D.A., Ugwu O.P.-C., Egba S.I., Agu P.C. (2025). Metabolomics-driven standardization of herbal medicine: Advances, applications, and sustainability considerations. Nat. Prod. Commun..

[99] Shen Y., Du H., Liu F., Gou X., Tao Y., Lan X., Zhang J., Yin Y., Li Q., Fan G. (2026). Quality control of herbal medicine based on analytical techniques and machine learning: Current advances and future perspectives. Phytochem. Anal..

[100] Arthika S., Mahalakshmi S., Ancy A., Vijayaragavan S.P., Kumar A.S., Arulselvi S., Dhinakar P., Christodoss P.R. (2025). MED-PLANT-XAI: An explainable deep learning and Flask information retrieval system for medicinal plant identification via CNN. Proceedings of the Global Conference on Sustainable Energy Systems, Smart Electronics and Intelligent Computing (GCSESEIC 2025).

[101] Kellogg J.J., Jordan R.T., Ranaweera M.M., Custer K., Anez S.G., Bendlin J., Chacon F.T., Chen X. (2026). Cultivar to chemotype: Characterizing complex botanicals with mass spectrometry metabolomics. Nat. Prod. Rep..

[102] Ezenarro J., Schorn-García D. (2025). How are chemometric models validated? A systematic review of linear regression models for NIRS data in food analysis. J. Chemom..

[103] Kirova G.K. (2026). Advances in analytical methods for quality control and authentication of nutraceuticals: A comprehensive review. Nutraceuticals.

[104] Schmidt S., Schindler M., Eriksson L. (2022). Block-wise exploration of molecular descriptors with multi-block orthogonal component analysis (MOCA). Mol. Inform..

[105] Homobono Brito de Moura P., Leleu G., Da Costa G., Marti G., Pétriacq P., Valls Fonayet J., Richard T. (2025). Integrating NMR and MS for improved metabolomic analysis: From methodologies to applications. Molecules.

[106] Urumarudappa S.K.J., Bommuluri V., J S., Chaturvedi S., Mittal A.K., Zhang Y., Chang P., Swanson G. (2025). Authentication methods for phytochemicals (botanicals) in plant extracts and dietary supplements. J. Diet. Suppl..

[107] AOAC INTERNATIONAL (2012). Guidelines for validation of botanical identification methods. J. AOAC Int..

[108] Kinghorn A.D., Falk H., Gibbons S., Asakawa Y., Liu J.-K., Dirsch V.M. (2023). Progress in the Chemistry of Organic Natural Products 122.

[109] Simmler C., Graham J.G., Chen S.N., Pauli G.F. (2018). Integrated analytical assets aid botanical authenticity and adulteration management. Fitoterapia.

[110] Ji P., Yang X., Zhao X. (2024). Application of metabolomics in quality control of traditional Chinese medicines: A review. Front. Plant Sci..

[111] Wallace E.D., Todd D.A., Harnly J.M., Cech N.B., Kellogg J.J. (2020). Identification of adulteration in botanical samples with untargeted metabolomics. Anal. Bioanal. Chem..

[112] Avoiding Products Contaminated with Hidden Ingredients. U.S. Food and Drug Administration (FDA). https://www.fda.gov/drugs/medication-health-fraud/avoiding-products-contaminated-hidden-ingredients.

[113] RASFF—Food Safety. European Commission. https://food.ec.europa.eu/food-safety/rasff_en.

[114] Wishart D.S., Guo A., Oler E., Wang F., Anjum A., Peters H., Dizon R., Sayeeda Z., Tian S., Lee B.L. (2022). HMDB 5.0: The human metabolome database for 2022. Nucleic Acids Res..

[115] Aisporna A., Benton H.P., Chen A., Derks R.J.E., Galano J.M., Giera M., Siuzdak G. (2022). Neutral loss mass spectral data enhances molecular similarity analysis in METLIN. J. Am. Soc. Mass Spectrom..

[116] Ferry-Dumazet H., Gil L., Deborde C., Moing A., Bernillon S., Rolin D., Nikolski M., de Daruvar A., Jacob D. (2011). MeRy-B: A web knowledgebase for the storage, visualization, analysis and annotation of plant NMR metabolomic profiles. BMC Plant Biol..

[117] Elapavalore A., Kondić T., Singh R.R., Shoemaker B.A., Thiessen P.A., Zhang J., Bolton E.E., Schymanski E.L. (2023). Adding open spectral data to MassBank and PubChem using open-source tools to support non-targeted exposomics of mixtures. Environ. Sci. Process. Impacts.

[118] Cabrera Allpas R., Li D.W., Choo M., Lee K., Bruschweiler-Li L., Brüschweiler R. (2025). COLMAR1d2d: Synergistic combination of 1D with 2D NMR for enhanced high-throughput identification and quantification of metabolites in complex mixtures. Anal. Chem..

[119] Poynton E.F., van Santen J.A., Pin M., Contreras M.M., McMann E., Parra J., Showalter B., Zaroubi L., Duncan K.R., Linington R.G. (2025). The Natural Products Atlas 3.0: Extending the database of microbially derived natural products. Nucleic Acids Res..

[120] Dictionary of Natural Products. Routledge/CRC Press. https://www.routledge.com/go/the_dictionary_of_natural_products.

[121] Sud M., Fahy E., Cotter D., Azam K., Vadivelu I., Burant C., Edison A., Fiehn O., Higashi R., Nair K.S. (2016). Metabolomics Workbench: An international repository for metabolomics data and metadata, metabolite standards, protocols, tutorials and training, and analysis tools. Nucleic Acids Res..

[122] Haug K., Salek R.M., Conesa P., Hastings J., de Matos P., Rijnbeek M., Mahendraker T., Williams M., Neumann S., Rocca-Serra P. (2013). MetaboLights—An open-access general-purpose repository for metabolomics studies and associated meta-data. Nucleic Acids Res..

[123] Yurekten O., Payne T., Tejera N., Amaladoss F.X., Martin C., Williams M., O’Donovan C. (2024). MetaboLights: Open data repository for metabolomics. Nucleic Acids Res..

[124] Spicer R.A., Salek R., Steinbeck C. (2017). Compliance with minimum information guidelines in public metabolomics repositories. Sci. Data.

[125] Abraham E.J., Kellogg J.J. (2021). Chemometric-guided approaches for profiling and authenticating botanical materials. Front. Nutr..

[126] AOAC INTERNATIONAL (2023). Guidelines for dietary supplements and botanicals. Official Methods of Analysis of AOAC INTERNATIONAL.

[127] Eurachem. http://www.eurachem.org.

[128] de Jonge N.F., Mildau K., Meijer D., Louwen J.J.R., Bueschl C., Huber F., van der Hooft J.J.J. (2022). Good practices and recommendations for using and benchmarking computational metabolomics metabolite annotation tools. Metabolomics.

[129] Zhou Z., Luo M., Zhang H., Yin Y., Cai Y., Zhu Z.J. (2022). Metabolite annotation from knowns to unknowns through knowledge-guided multi-layer metabolic networking. Nat. Commun..

[130] Ludwig M., Nothias L.F., Dührkop K., Koester I., Fleischauer M., Hoffmann M.A., Petras D., Vargas F., Morsy M., Aluwihare L. (2020). Database-independent molecular formula annotation using Gibbs sampling through ZODIAC. Nat. Mach. Intell..

[131] Dührkop K., Shen H., Meusel M., Rousu J., Böcker S. (2015). Searching molecular structure databases with tandem mass spectra using CSI:FingerID. Proc. Natl. Acad. Sci. USA.

[132] Dührkop K., Nothias L.F., Fleischauer M., Ludwig M., Hoffmann M.A., Rousu J., Dorrestein P.C., Böcker S. (2020). Classes for the masses: Systematic classification of unknowns using fragmentation spectra. bioRxiv.

[133] Morshedloo M.R., Salami S.A., Nazeri V., Maggi F., Craker L. (2018). Essential oil profile of oregano (*Origanum vulgare* L.) populations grown under similar soil and climate conditions. Ind. Crops Prod..

[134] Jafari Khorsand G., Morshedloo M.R., Mumivand H., Emami Bistgani Z., Maggi F., Khademi A. (2022). Natural diversity in phenolic components and antioxidant properties of oregano (*Origanum vulgare* L.) accessions, grown under the same conditions. Sci. Rep..

